# Genome-wide investigation of microRNAs and expression profiles during rhizome development in ginger (*Zingiber officinale* Roscoe)

**DOI:** 10.1186/s12864-021-08273-y

**Published:** 2022-01-13

**Authors:** Haitao Xing, Yuan Li, Yun Ren, Ying Zhao, Xiaoli Wu, Hong-Lei Li

**Affiliations:** 1 College of Landscape Architecture and Life Science/Institute of Special Plants, Chongqing University of Arts and Sciences, Chongqing, 402168 China; 2 Chongqing Key Laboratory of Economic Plant Biotechnology, Chongqing University of Arts and Sciences, Chongqing, 402168 China; 3 Research Center for Terrestrial Biodiversity of the South China Sea, Institute of Tropical Agriculture and Forestry, Hainan University, Haikou, 570228 Hainan China

**Keywords:** *Zingiber officinale* Roscoe, Genome-wide, miRNA, Rhizome, growth and development

## Abstract

**Background:**

MicroRNAs (miRNAs) are endogenous, non-coding small functional RNAs that govern the post-transcriptional regulatory system of gene expression and control the growth and development of plants. Ginger is an herb that is well-known for its flavor and medicinal properties. The genes involved in ginger rhizome development and secondary metabolism have been discovered, but the genome-wide identification of miRNAs and their overall expression profiles and targets during ginger rhizome development are largely unknown. In this study, we used BGISEQ-500 technology to perform genome-wide identification of miRNAs from the leaf, stem, root, flower, and rhizome of ginger during three development stages.

**Results:**

In total, 104 novel miRNAs and 160 conserved miRNAs in 28 miRNA families were identified. A total of 181 putative target genes for novel miRNAs and 2772 putative target genes for conserved miRNAs were predicted. Transcriptional factors were the most abundant target genes of miRNAs, and 17, 9, 8, 4, 13, 8, 3 conserved miRNAs and 5, 7, 4, 5, 5, 15, 9 novel miRNAs showed significant tissue-specific expression patterns in leaf, stem, root, flower, and rhizome. Additionally, 53 miRNAs were regarded as rhizome development-associated miRNAs, which mostly participate in metabolism, signal transduction, transport, and catabolism, suggesting that these miRNAs and their target genes play important roles in the rhizome development of ginger. Twelve candidate miRNA target genes were selected, and then, their credibility was confirmed using qRT-PCR. As the result of qRT-PCR analysis, the expression of 12 candidate target genes showed an opposite pattern after comparison with their miRNAs. The rhizome development system of ginger was observed to be governed by miR156, miR319, miR171a_2, miR164, and miR529, which modulated the expression of the *SPL*, *MYB*, *GRF*, *SCL*, and *NAC* genes, respectively.

**Conclusion:**

This is a deep genome-wide investigation of miRNA and identification of miRNAs involved in rhizome development in ginger. We identified 52 rhizome-related miRNAs and 392 target genes, and this provides an important basis for understanding the molecular mechanisms of the miRNA target genes that mediate rhizome development in ginger.

**Supplementary Information:**

The online version contains supplementary material available at 10.1186/s12864-021-08273-y.

## Introduction

MicroRNAs (miRNAs) are endogenous noncoding small RNA molecules with a length of 20–24 nucleotides (nt). In the past few decades, it has been shown that many miRNAs are evolutionarily conserved across diverse plant species and play essential roles in the post-transcriptional regulation of growth and development, biotic/abiotic responses, and other important biological processes of plants [1, 2].

As an evolutionarily conserved microRNA, miR156 regulates the vegetative phase transition by modulating the expression of a subset of *SQUAMOSA PROMOTER BINDING PROTEIN-LIKE* (*SPL*) genes in diverse flowering plants [3]. miR164 and its target, *NAC DOMAIN CONTAINING PROTEIN 1* (*NAC1*), participate in the formation of lateral roots in Arabidopsis and maize [4, 5]. miR172 regulates floral development and flowering time through the repression of AP2 genes, and has been reported in a variety of plant species, including Arabidopsis, barley, soybean, and rice [6–9].

It has been shown that a crucial regulatory module, miR396-GRF/GIF, controls the growth of multiple tissues and organs in Arabidopsis [10–12], tomato [13], and rice [14]. Yang et al. [15] reported that the GpmiR166b-*GpECH2* module participated in the stem-to-rhizome transition and probably promoted cell expansion by indole-3-butyric acid (IBA)-to-indole-3-acetic acid (IAA) conversion, and the GpmiR166e-*GpSGT*-like module promoted rhizome formation in *Gynostemma pentaphyllum*. In rice, overexpressing OsmiR397a and OsmiR397b transgenic lines show strongly nodding panicles in comparison with wild-type plants. Further molecular and genetic results determined that miR397 leads to an increase in grain yield by the downregulation of its target gene *LAC*, a regulator involved in the sensitivity of plants to brassinosteroids [16]. In turmeric, it was observed that miR156 and miR5015 were involved in rhizome growth and development, while miR5021 participated in terpenoid and isoquinoline alkaloid biosynthesis [17].

In 1993, the first miRNA, lin-4, was identified from *Caenorhabditis elegans* [18]. In plants, miRNAs were first identified from Arabidopsis [19]. Following that, some miRNAs were revealed in plants by cloning and bioinformatics prediction [20–23]. The cloning method is more laborious and can only be used to identify miRNAs on a small scale. High-throughput sequencing technology not only yields abundant reads, but it can also detect the expression of minimally abundant small RNAs [24]. Therefore, high-throughput sequencing technology has become the technique of choice for discovering miRNAs [24]. In 2005, Arabidopsis miRNAs were identified using high-throughput sequencing technology [25]. Since then, thousands of miRNAs have been characterised from different species using this technology [26–29]. Based on the miRBase database (https://www.mirbase.org/; Release 22.1, Oct 2018), a total of 38,589 mature miRNA sequences from 271 different species (ranging from viruses to humans) have been currently identified. However, few studies have been conducted on the identification of miRNAs in ginger.

Ginger (*Zingiber officinale* Rosc.), belonging to the genus *Zingiber* in the family Zingiberaceae, is recognized not only as a spice but also as a traditional Eastern medicine for treating chronic inflammation, such as in asthma, rheumatoid arthritis, and other ailments [30]. Gingerols and other flavor compounds are derived from the rhizome of this plant, as well as valuable research materials for abnormal stem identification, growth, and development [31]. Despite their importance, very few genes have been identified from ginger rhizomes. Moreover, very little is known about the miRNAs involved in gingers rhizome identity, growth, and development in general.

Koo et al. [32] analyzed the expressed sequence tags (ESTs) from two ginger lines (white ginger and yellow ginger) and investigated the expression of the corresponding genes in the rhizome. They showed that AUX/IAA and MADS box protein play a crucial role in rhizome initiation and development. Singh et al. [33] used a homology search-based computational approach for identifying miRNAs in *Z. officinale*. They used the ESTs of *Z. officinale*, which may have lost some important information. However, how ginger rhizome undergoes initiation and expansion is still a mystery. With the accomplishment of ginger genome assembly and annotation by our group [34], we performed a more accurate genome-wide analysis of miRNAs and their targets using high-throughput sequencing technology.

In the present study, therefore, high-throughput sequencing and bioinformatics analysis was employed to identify miRNAs genome-wide and find tissue-specific expression profiles. We then conducted integrated analyses of miRNA and their targets to investigate the molecular mechanism underlying rhizome identity, growth, and development. We also expect that our findings will broaden our understanding of the abnormal development of plant stems.

## Results

### Library construction and sequencing of small RNAs

To identify the miRNAs in ginger, total RNAs were extracted from leaf, stem, root, flower, and rhizome in three different development stages: 1st I_d (first internode), 2nd I_d (second internode), 3rd I_d (third internode) of ginger and then used to construct 7 small RNA libraries. Then, the 7 small RNA libraries were sequenced using the BGI-tech Standard Small RNA sequencing platform and underwent bioinformatics analysis. A total of 28,804,171 raw tag counts from the leaf, 29,640,807 from the root, 29,630,116 from the 1st I_d of the rhizome, 29,084,284 from the 2nd I_d of the rhizome, 28,757,890 from the 3rd I_d of the rhizome, 29,115,222 from the stem, and 28,991,447 from the flower were obtained. After removal of adaptors, low quality reads, and contaminants, 26,648,833 clean tag counts from the leaf, 24,815,451 from the root, 26,324,400 from the 1st I_d of rhizome, 26,825,995 from the 2nd I_d of rhizome, 26,253,384 from the 3rd I_d of rhizome, 26,823,604 from the stem, and 26,988,071 from the flower were kept (Supplementary Table S1).

**Fig. 1:**
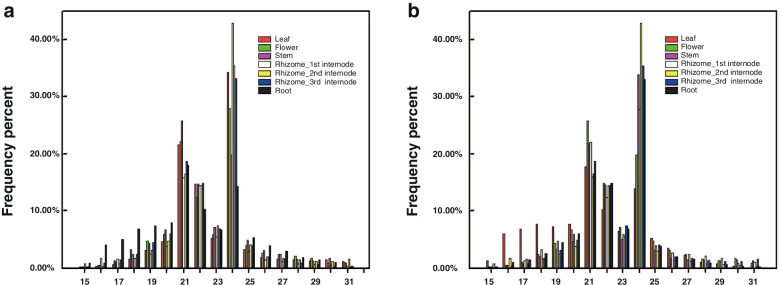
The length distribution of the clean and unique reads from leaf, stem, root, flower, and rhizome (1st I_d, 2nd I_d, and 3rd I_d) of *Z. officinale*. **a** Clean reads; **b** unique reads

The clean reads and unique reads of the 7 tissues were subjected to an analysis of the size distribution, as shown in Fig. 1. The lengths of the small RNAs in 7 samples were 15 to 31 nt. The 21 nt class was the most abundant in the stem, followed by 24, 22, and 20 nt classes (Fig. 1a). In the rhizome, 24 nt small RNAs were the most frequent, followed by 21, 22, and 23 nt. The 24-nt peak was found to be dominant at a unique read level in all 7 samples except for the flower and leaf, while the peak of 21 nt small RNAs was found in the flower and leaf (Fig. 1b).

### Conserved and novel miRNAs in ginger

To analyze the population of conserved miRNAs in ginger, the miRNA sequences of the 7 libraries were searched against the currently known mature miRNAs from other plants and ginger RNA sequences. As a result, a total of 160 conserved miRNA precursors were identified from 7 sRNA datasets (Supplementary Table S2). All these conserved miRNAs of ginger belong to 28 miRNA families, and at least one precursor was identified in most miRNA families. Among them, MIR396 was the largest family identified with 16 members, followed by MIR319 with 14 members. There were 13 members in the MIR156, MIR169, MIR171, and MIR166 families. However, several miRNA families were found to possess only one precursor, including MIR162, MIR165, MIR477, MIR5179, MIR528, MIR5523, and MIR5532.

The sRNA sequencing results indicated that the clean counts of conserved miRNAs ranged from 1 up to more than 10,000 in 7 samples. Of all conserved miRNAs, the clean counts of MIR159, MIR160, and MIR395 exceeded 10,000 in one tissue. The clean counts of ten miRNA families (MIR156, MIR164, MIR166, MIR167, MIR168, MIR171, MIR319, MIR396, MIR398, and MIR528) ranged from 10,000 to 50,000 at least in one tissue. However, the other miRNA families (MIR157, MIR160, MIR162, MIR165, MIR169, MIR172, MIR390, MIR393, MIR395, MIR399, MIR408, MIR5523, MIR5532, and MIR845) had fewer than 5000 reads in all 7 tissues (Supplementary Table S2).

In addition to the conserved miRNAs, some novel miRNA sequences were also found in the 7 libraries from the remaining sRNA sequences after removal of the tRNAs, rRNAs, snoRNAs, snRNAs, and known miRNAs. According to the criteria for plant miRNAs, a total of 104 novel miRNAs were identified in 7 libraries. The length of novel miRNA sequences ranged from 19 to 30 nt. However, the sequences of most novel miRNAs were 24 nt in length, followed by 25, 21, and 22 nt. The length of pre-miRNAs ranged from 52 to 1439 nt. The average minimum folding free energy value of the hairpin structures was − 160.719 kcal/mol in *Z. officinale* Roscoe (Supplementary Table S3). The secondary structures of 104 novel miRNA precursors are shown in Supplementary Fig. S1. The actual tag count span of novel miRNAs was large in the 7 libraries, varying from 0 to 74,105. Among these novel miRNAs, novel miR110, miR177, miR192, miR22, miR23, miR3, miR7, and miR90 accounted for more than 2000 counts in some of the 7 libraries.

**Fig. 2:**
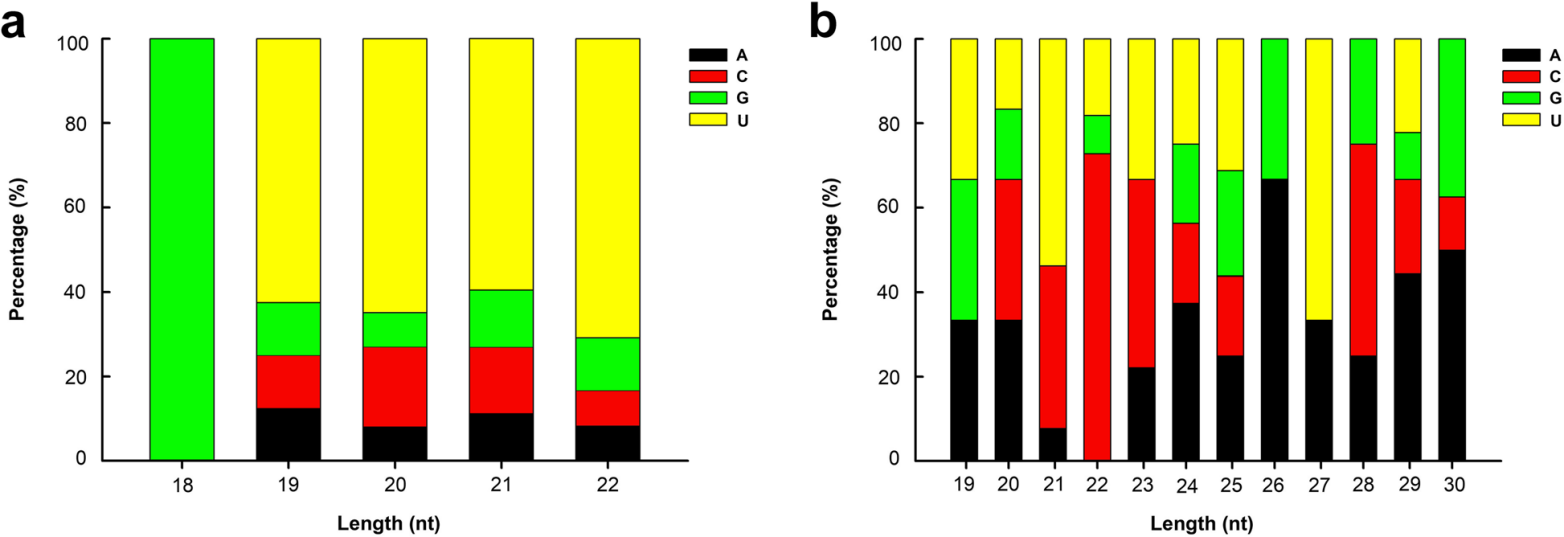
Statistics of the first base of conserved and novel miRNAs

The first nucleotide at the 5′-terminus of mature miRNA had a strong base preference. The conserved miRNAs had a predominance of U at the 5′-terminus except for the 18-nt clade (Fig. 2a). These results are in agreement with previous reports. We also found that miRNAs with a length of 22 nt possessed the lowest number of G bases, and the largest base species at the 5′-terminus existed in 21-nt miRNAs, which is in accordance with the characteristics of miRNA base preferences (Fig. 2a). In the conserved miRNA, uracil was the first preferred base of the 5′-terminus of the 21 potential novel miRNA sequences (Fig. 2b). As with the novel miRNAs, additional base species at the 5′-terminus were found in miRNAs with lengths of 21 and 27 nt.

### miRNA expression profile in different tissues of ginger

miRNAs are known to play an important role in the growth and development of plants. Several miRNA genes are differentially expressed in specific tissues/organs. The expression profiles of miRNAs were normalized by transcripts per million (UMI) for further comparative analysis. Based on a criteria of |log2 (fold change)| ≥ 1 and qvalue< 0.005, differential expression patterns of 90 of the most abundant conserved miRNAs and 62 novel miRNAs in 7 libraries were analyzed.

**Fig. 3:**
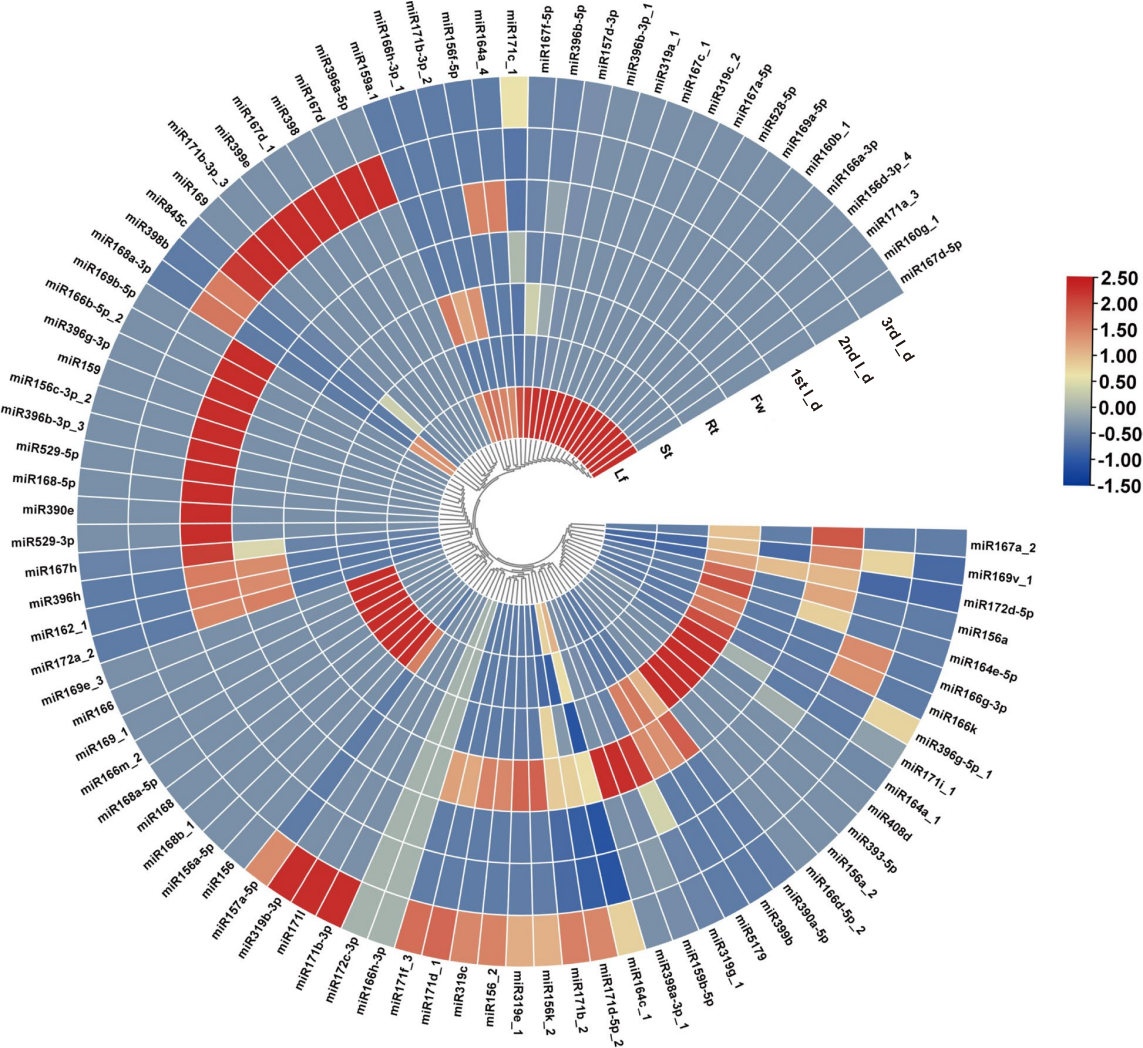
Expression profiles of conserved miRNAs in 7 different tissues. Fw: flower; Lf: leaf; St: stem; Rt: root; Rz_1: Rhizome 1st I_d; Rz_2: Rhizome 2nd I_d; Rz_3: Rhizome 3rd I_d

We found that 17, 9, 8, 4, 13, 8, and 3 conserved miRNAs exhibited significant tissue-specific expression patterns in leaf, stem, root, flower, 1st I_d, 2nd I_d, and 3rd I_d of the rhizome, respectively (Fig. 3). In leaves, one or more isoforms of miR156, miR157, miR160, miR167, miR169, miR171, miR319, miR396, and miR528 were abundant, indicating that these miRNAs were involved in leaf development. miR167h, miR529-3p, miR529-5p, miR166b-5p-2, miR156c-3p-2, miR396b-3p-3, miR168-5p, miR390e, and miR396g-3p were more abundant in the flower than in other tissues. The expression of miR166k, miR396g-5p_1, miR171i-1, miR164a_1, miR408d, miR393-5p, and miR156a_2 was upregulated in roots. We also detected 3 miRNA families with more abundance in the stem (miR166, miR166m_2, miR168a-5p, miR168, miR168b_1, miR169_1, and miR169e_3).

**Fig. 4:**
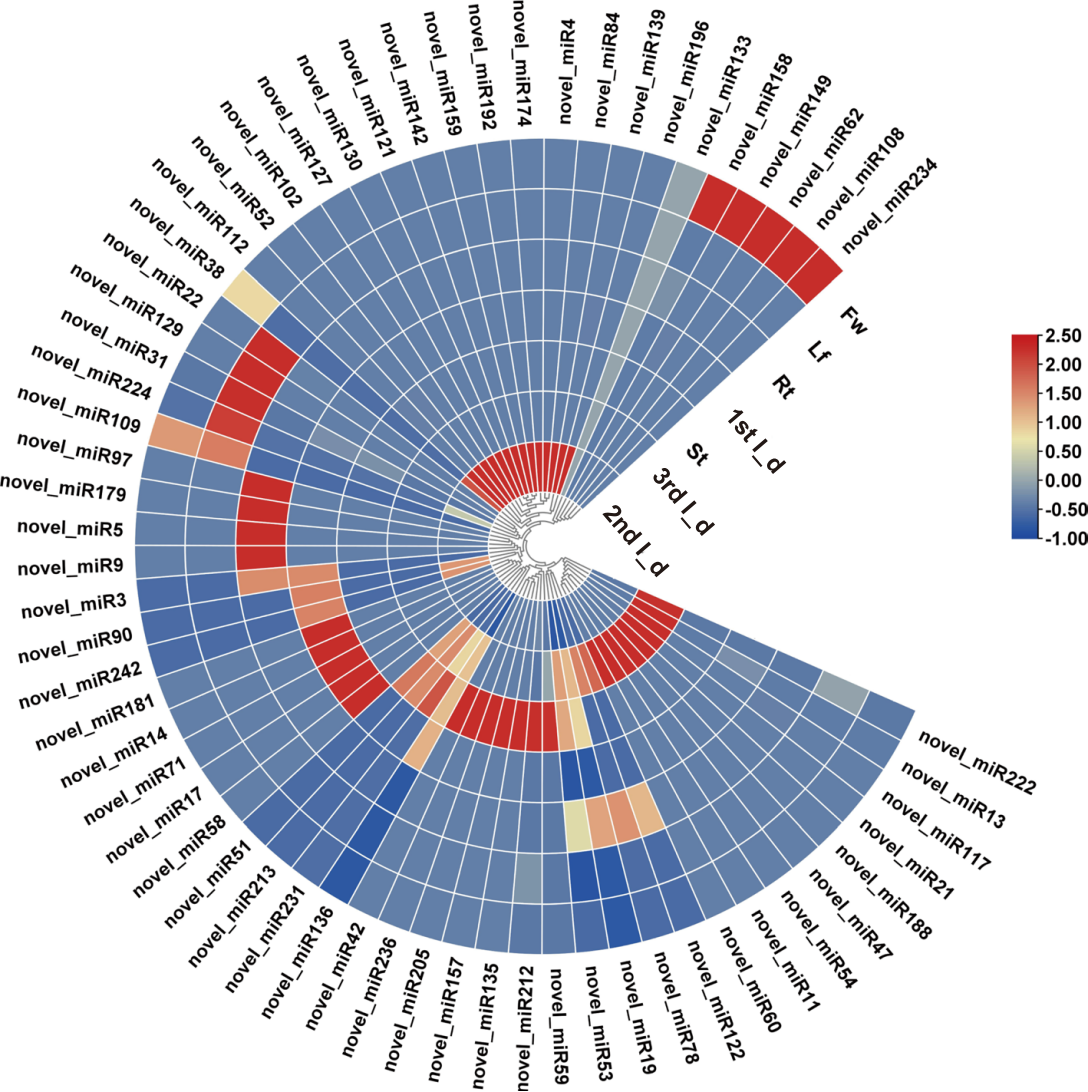
Expression profiles of novel miRNAs in 7 different tissues. Fw: flower; Lf: leaf; St: stem; Rt: root; Rz_1: Rhizome 1st I_d; Rz_2: Rhizome 2nd I_d; Rz_3: Rhizome 3rd I_d

We found that 5, 2, 4, 7, 2, 5, and 2 novel miRNAs exhibited significant tissue-specific expression patterns in the leaf, stem, root, flower, 1st I_d, 2nd I_d, and 3rd I_d of the rhizome, respectively (Fig. 4 and Supplementary Table S2). The expression levels of novel_miR234, novel_miR108, novel_miR62, novel_miR149, and novel_miR158 in the flower were higher than those in other tissues. Novel_miR22, novel_miR129, novel_miR31, and novel_miR224 were detected with higher expression in leaves. Novel_miR97, novel_miR179, novel_miR5, and novel_miR9 were more abundant in roots, whereas novel_miR181, novel_miR14, novel_miR71, novel_miR17, and novel_miR58 were more abundant in stems.

### Cluster analysis of differentially expressed miRNAs in ginger rhizome development

Among three different rhizome development stages (1st I_d, 2nd I_d, and 3rd I_d), approximately 22 conserved miRNAs were downregulated, and 8 miRNAs were conserved in the 2nd I_d in comparison with the 1st I_d. Additionally, 8 conserved miRNAs were downregulated, and 15 conserved miRNAs were upregulated in the 3rd I_d in comparison with the 2nd I_d (Supplementary Fig. S2). For example, the expression ratios (2nd/1st I_d) of miR164k and miR398 were 91.39 and 37.15, respectively. However, the 3rd/2nd I_d ratios of the two miRNAs were 0.011 and 0.0.026, respectively. Interestingly, miR169v_1 was gradually decreased during rhizome development.

The novel miRNAs were similar to the conserved miRNAs in that approximately 12 novel miRNAs were upregulated and 7 were downregulated in the 2nd I_d as compared to the 1st I_d stage. There were no changes in the other 14 novel miRNAs (Supplementary Table S3). Of the novel miRNAs, 12 were downregulated, while 11 novel miRNAs were upregulated, and most of them were upregulated in the 3rd I_d compared to the 2nd I_d. Novel miR90 was gradually decreased in rhizome development. There was higher expression of novel miR13 in the 1st I_d and decreased expression in the 2nd I_d stage, which then increased once more in the 3rd I_d. These results suggest that these miRNAs may play a major role in the rhizome growth and development of ginger (Supplementary Fig. S3).

### MiRNA targets prediction in ginger rhizome

**Fig. 5:**
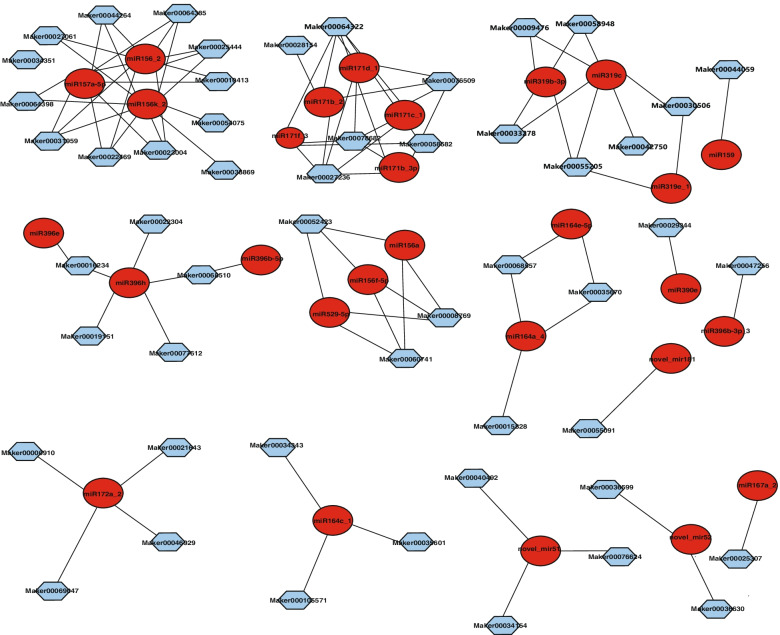
The regulatory network of miRNA and targeted genes in 3 rhizome developing stages. A red circle denotes miRNA, and a cyan hexagon denotes the predicted targets

Plant miRNAs function with a mechanism of suppressing the translation of target genes or cleaving target mRNAs in diverse vital biological processes. The putative target gene prediction analyses showed that 365 target genes for 44 conserved miRNA families and 27 target genes for 8 novel miRNAs were predicted (Fig. 5 and Supplementary Table S4). It was found that the majority of the target genes for conserved miRNAs were transcription factors. A number of these target genes were conserved between ginger and other plants, such as *MYB*, *TCP*, auxin response factor (*ARF*), LOB domain-containing protein, meristem (*NAM*) protein, *DCL1*, NAC domain transcription factor, *AGO1*, *APETALA2*, ATP-dependent RNA helicase, squamosa promoter-binding proteins (*SPL*), and growth-regulating factors (*GRF*), which are involved in various aspects of secondary metabolic substance biosynthesis and the tissue growth and development of plants. However, some predicted target genes of several conserved miRNAs in ginger were different from those in other plants, including pirin-like protein, (MIR156) homeobox-leucine zipper protein (MIR166), and TIFY (MIR167) (Supplementary Table S4). It was mainly predicted that the target genes of novel miRNAs were *MYB* transcriptional factor, serine/threonine-protein kinase, multidrug resistance protein, F-box/LRR-repeat protein, polyol transporter, and LRR receptor-like serine/threonine-protein kinase (Supplementary Table S4).

### Gene ontology (GO) of miRNA target genes in ginger rhizome

**Fig. 6:**
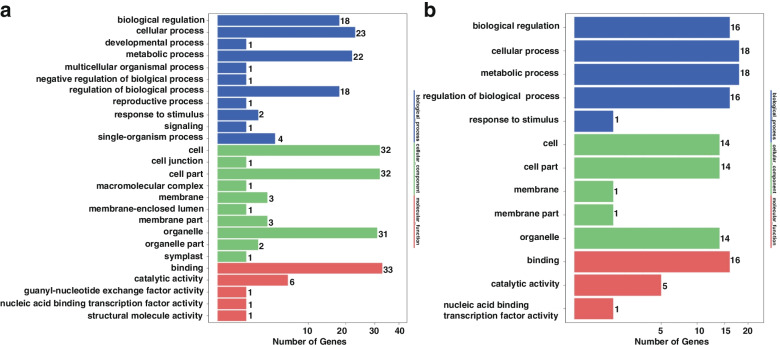
GO analysis of miRNA targets involving rhizome development. **a** The GO analysis between the 2nd I_d and 1st I_d; **b** the GO analysis between the 3rd I_d and 2nd I_d. The ordinate is classified as GO, with the number of genes on the right side of the terms

In order to further analyze the biological function of miRNA targets in rhizome development, GO analysis was implemented for all the miRNA target genes and differentially expressed genes between the 2nd and 1st I_d, and 3rd and 2nd I_d, respectively (Fig. 6). As shown in Fig. 6a, there are some specific functions with large inferences in the number of target genes in the three GO terms, such as metabolic regulation of biological process, cell process, cell part, organelle in cellular components, and binding in molecular function.

Comparing the 3rd and 2nd I_d, some specific functions with large differences were found in the number of target genes in the three GO terms, such as nucleoid in cellular components and cell junction (Fig. 6b). Most of the miRNAs were downregulated in the 2nd I_d and then upregulated in the 3rd I_d, and thus, the significant difference in the number of genes for these secondary functions was probably caused by cell proliferation and expansion.

### The expression of miRNAs and their target genes during ginger rhizome development

**Fig. 7:**
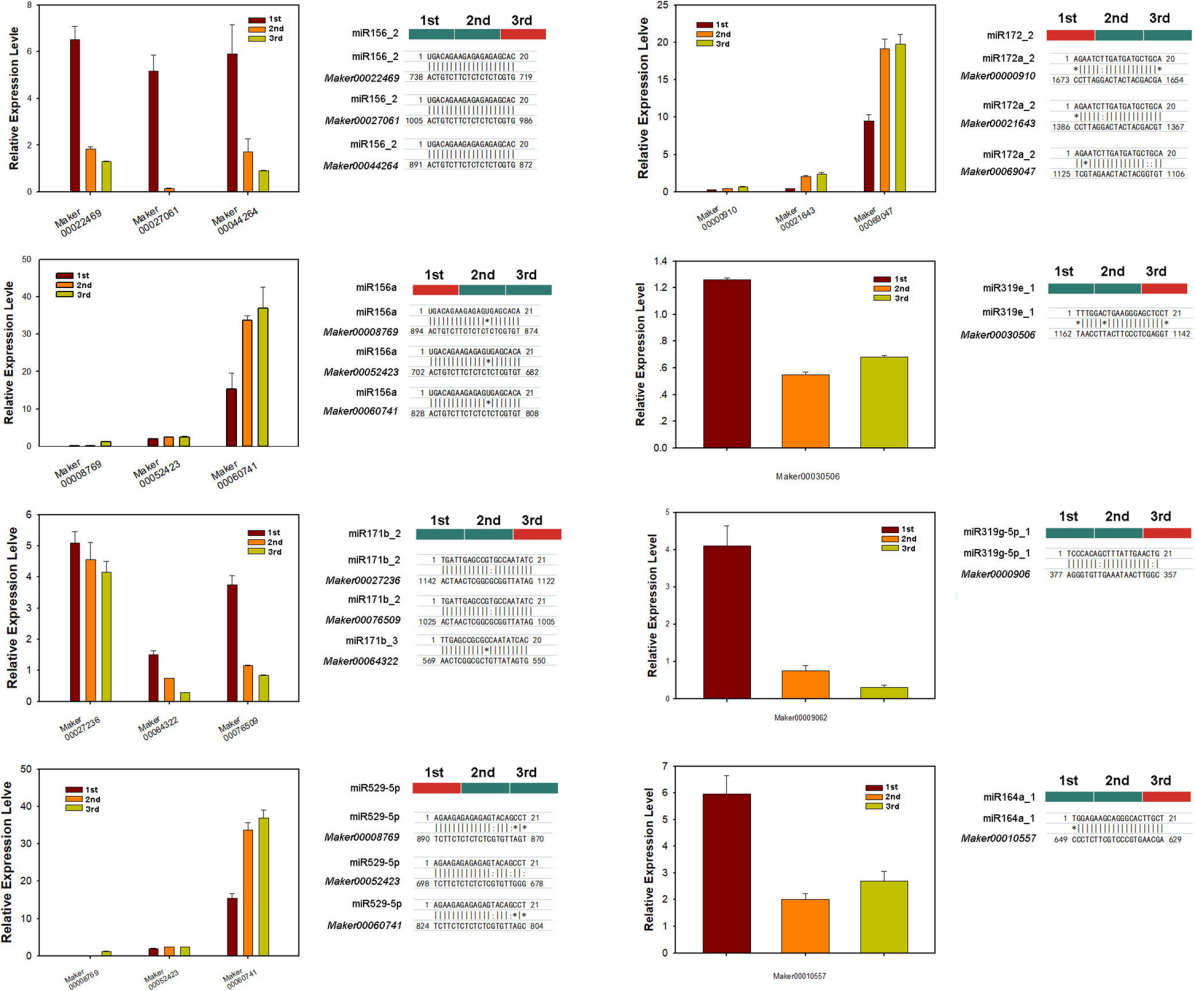
Validation of target genes by miRNAs using qRT-PCR

To better understand the biological function of miRNAs in the development of ginger rhizome, miRNA target genes were predicted and are shown in Supplementary Table S4. Many studies have shown that miRNAs negatively regulate target gene expression in the regulation of plant growth and development. To validate the regulation of target expression by miRNA, 8 miRNAs (miR156_2, miR156a, miR171b_2, miR172_2, miR164a_1, miR319e_1, miR319g-5p_1, and miR529) were selected, and their target gene expression in the 1st I_d, 2nd I_d, and 3rd I_d of ginger was detected using qRT-PCR analysis (Fig. 7).

*Maker00022469*, *Maker00027061*, and *Maker000044264* are members of the SPL family and were predicted targets of miR156_2. These three genes were found more abundantly in the rhizome 1st I_d, and then decreased in the 2nd and 3rd I_d stage, while tmiR156_2 exhibited the opposite expression pattern in comparison with these targets. *Maker00000910*, *Maker00021643*, and *Maker00069047* were predicted targets of miR171a_2. miR319e-1 and miR319g-5p_1 were significantly upregulated during the enlargement of ginger rhizome, whereas their target genes, which encode *MYB33*-like (*Maker00030506*) *and GRF4-*like (*Maker00009062*), were significantly downregulated, respectively. In contrast, miR529-5p and miR172_2 were significantly down-regulated during the development of ginger rhizome, and their targets were significantly up-regulated. Like miR156a, miR529-5p also targeted SPL13 transcription factor genes (*Maker00008769*, *Maker00052423*, and *Maker00060741*) with different target sites.

## Discussion

miRNAs are conserved across plant species, which offers the possibility of miRNA identification through homologue identification in a new plant species [33, 35]. In the current study, a high-throughput sequencing approach was used to search for conserved and novel miRNAs in seven different tissues of ginger. Initially, based on a homology search approach, 160 conserved miRNAs belonging to 28 miRNA families and 104 novel miRNAs were identified according to the criteria for plant miRNAs. Position-specific dominance of length variation and of miRNAs were analysed at the sequence level. The length of miRNAs ranged from 15 to 31, whereas the most abundant miRNAs were 21 and 24 nt in length. Like other plant species, uracil was the dominant base at the first position of mature miRNAs [19]. We calculated the MFE to assess the stability of the secondary structure of novel miRNAs. The average MFE value for ginger novel miRNAs was − 160.719 (kcal/mol). In contrast with Arabidopsis miRNAs, the majority of the novel miRNA sequences for ginger were lower than those of shuffled sequences, which indicates a high tendency for stable secondary structure.

**Fig. 8:**
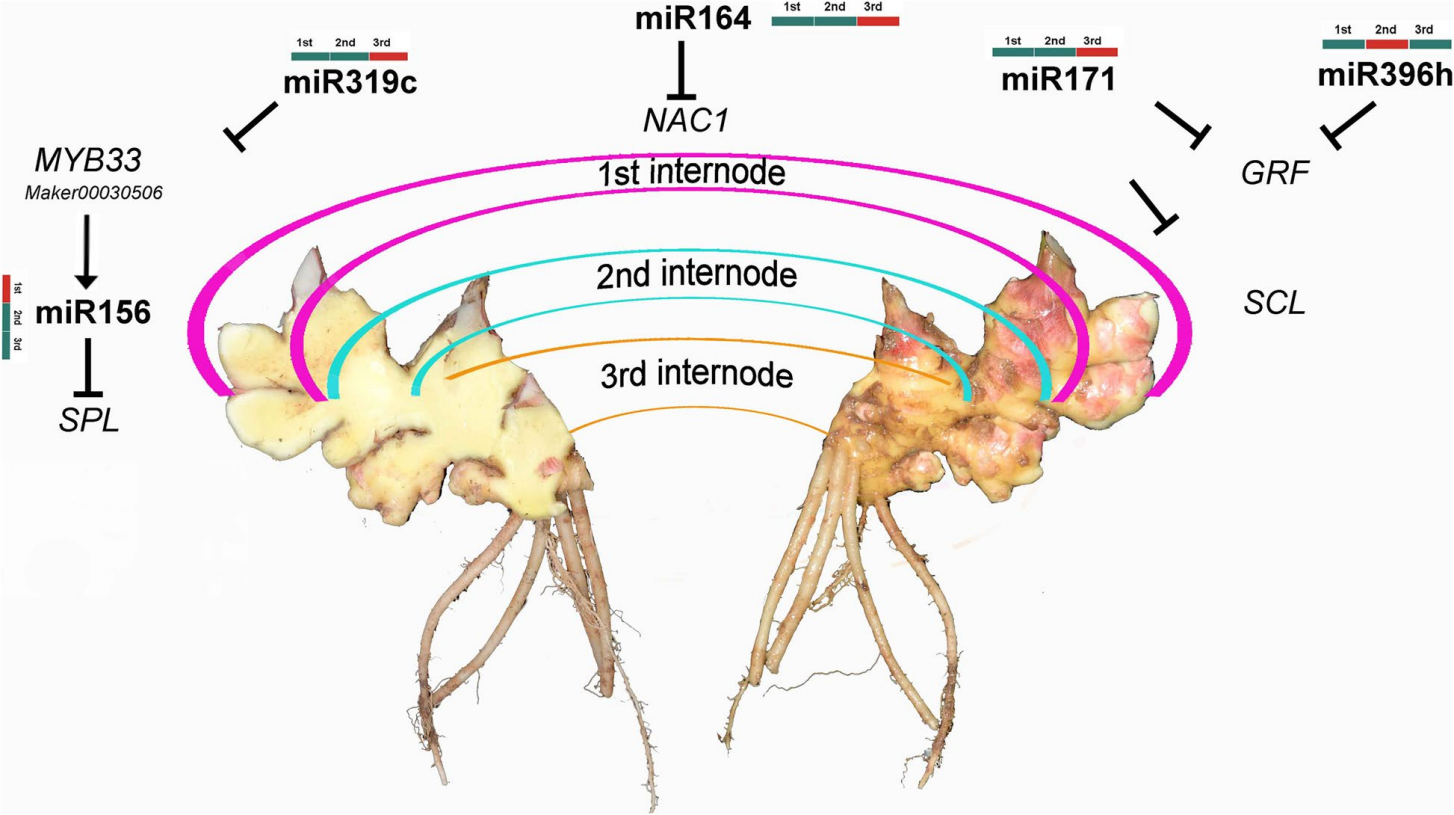
Hypothetic model of rhizome development and miRNA-mediated regulation in ginger

In the past decade, increasing amounts of evidence have demonstrated that miRNAs play essential roles in post-transcriptional gene regulation of base pairing with their complementary mRNA targets, and they especially prefer to target transcription factors in plants [1]. Target prediction of ginger miRNAs was performed for coding transcripts, and a total of 2953 mRNA targets was identified. Networking analyses suggested that miR156 and miR5021 are responsible for most of the co-regulated targets. In the case of ginger, it was found that miR5021 was involved in the co-regulation of many targets. It was observed that miR156, miR319, miR171a_2, miR164, and miR529 were differently expressed during the rhizome development system. These miRNAs were found to target *SPL*, *MYB*, *GRF*, *SCL*, and *NAC* genes, respectively (Figs. 5 and 8).

miR156 family members are well studied and function across various plant developmental traits [36]. In Arabidoposis, miR156-*SPL*s module is considered as key molecular integrator in regulating the juvenile to adult phase transition [37]. Three homologs of *AthSPLs* in ginger including *Maker00008769* (*AthSPL3* homolog), *Maker00052423* (*AthSPL13* homolog), and *Maker00060741* (*AthSPL3* homolog) were identified as under negative regulation by miRNA156a. Interestingly, the expression level of miR156_2 was obviously increased during rhizome growth and development, and its targets *Maker00010413* (*AthCAT5* homolog), *Maker00022469* (*AthSPL9* homolog), *Maker00023004* (*AthSPL2* homolog), *Maker00025444* (*AthSPL2* homolog), *Maker00027061* (*AthSPL13* homolog), *Maker00031959* (*AthSPL9* homolog), *Maker00044264* (*AthSPL13* homolog), *Maker00064385* (*AthSPL13* homolog), and *Maker00064398* (*AthSPL2* homolog) also belong to the SPL gene family. This indicates that members of *SPL* family of ginger could have essential roles in rhizome development and were under negative regulation of miRNA156a and positively regulation of miR156_2. Furthermore, we found *Maker00064385* (*SPL13* homologue) was also targeted by miR529, but the target sites were different from those of miR156a.

The expression of miR319c was more abundant in the 1st I_d, and then it gradually decreased in the 2nd and 3rd rhizome development stages. One of its targets, *Maker00030506* (*MYB33* homolog), exhibited the opposite expression profile. In Arabidopsis, *MYB33* targeted by miR159 act as modifiers of the juvenile-to-adult transition by regulateing the expression of miR156 [38]. In the current study, we found that miR319 expression level was very low, whereas *Maker00030506* (*MYB33* homolog) was more abundantly expressed in the juvenile stage. Along with the development of rhizomes, miR319 expression was increased, and the expression of *Maker00030506* and miR156a were decreased during the same stage. These indicate that the miR319c/Maker0030506 regulator may be involved in rhizome development. The other target of miR319 was *Maker00009476*, which is the *TCP4* homolog of Arabidopsis. Schommer et al. [39] reported that a small increase in *TCP4* activity has an immediate impact on leaf cell number by significantly reducing cell proliferation. In this study, we proved that the *TCP4*-like gene (*Maker00009476*) was found in greater abundance in Rz_1 and was downregulated in Rz_2 and Rz_3. Thus, decreased expression of *Maker00009476* may promote the rhizome expansion of ginger. *Maker00055205* (*TCP24* homolog) modulates secondary cell wall thickening, which is negatively regulated by miR319. Along with rhizome development, the biosynthesis of the secondary cell wall was decreased. miR319/*Maker00055205* may be involved in rhizome fiber biosynthesis.

We also found that miRNA164 and miR172a were involved in rhizome development. *Maker00010557* (*NAC1* homolog) was negatively regulated by miR164. In Arabidopsis, *NAC1* was negatively regulated by miR164, which regulates lateral root formation [4]. *Maker00009062* and *Maker00009306* are homologs of *GRF4* in Arabidopsis, which coordinates cellulose biomass production and utilization. In the current study, we found that *Maker00009062* and *Maker00009306* were targeted by miR396h and miR172a. These two miRNAs were decreased in rhizome development. Wang et al. [40] demonstrated that *SCL6* was involved in regulating shoot branching. Negative expression of miR171 was found during rhizome development. It was found that nine *SCL6* homologous genes were negatively regulated by miR171 and decreased during rhizome development, indicating that miR171 and its targets could be involved in rhizome branching. Although, we found some tissue specific novel miRNAs were differently expressed during the rhizome development, their targets mainly were genes that coding uncharacterized proteins or that not involved in any GO categories (Supplementary Table S4). The functions of these novel miRNAs need to be further investigated.

## Conclusions

Ginger (*Zingiber officinale*), which belongs to the genus *Zingiber*, is known as a perennial herbaceous plant that grows from a rhizome. It is among the most important edible and medicinal crops in east and southeast Asia. To identify miRNAs in ginger and understand their potential functions, we constructed and sequenced 7 small RNA libraries from the flower, leaf, root, stem, and rhizome of *Zingiber officinale* Roscoe cultivar “LAIWU 2 J”. We subsequently identified 160 conserved miRNAs and 104 novel miRNAs from seven small RNA libraries through high-throughput sequencing, and predicted their putative targets using Target Finder software. This study presents a first report on the investigation of miRNAs and their targets involved in the rhizome development of ginger. Our findings may also be helpful in developing strategies aimed at enhancing abnormal stem production based on miRNAs. Indeed, further experiments are required to characterize the role of miRNAs in the regulation of rhizome development of this important edible and medicinal plant.

## Materials and methods

### Plant material and RNA preparation

The plants of the *Zingiber officinale* Roscoe cultivar ‘LAIWU 2 J’ were maintained by conventional cultivation and management in the greenhouse of the Chongqing University of Arts and Sciences. Root, leaves, stem, flowers, and 1st I_d, 2nd I_d and 3rd I_d of ginger rhizome were collected and immediately frozen in liquid nitrogen. Then, the frozen samples were stored at − 80 °C for future analysis. Total RNA was extracted from 7 samples using TRIzol® reagent (Invitrogen, USA) according to the manufacturer’s protocol. Finally, an Agilent 2100 Bioanalyzer (Agilent Technologies, USA) and 1% agarose gel electrophoresis were used to confirm the integrity of total RNA. The analysis of each library was repeated three times.

### Library construction and sequencing of small RNA

Small RNA libraries of root, leaves, stem, flowers, and rhizome were constructed following the methods described in Evers et al. (2015). Briefly, small RNA fragments 10–30 nt in length were purified using a 15% denaturing polyacrylamide gel, followed by ligating with 5′ and 3′ adapters. Adapter-ligated RNAs were reverse-transcribed by Superscript II reverse transcriptase (Invitrogen) and amplified by PCR. Then, approximately 20 μg products from each sample was sequenced using the BGISEQ-500 platform (BGI, Inc.; Shenzhen, China).

### Prediction of miRNAs and their targets

Chip adaptor sequences, low quantity reads, and contaminations were removed. Bowtie2 is used to map clean reads to the reference genome and to other sRNA databases. Then, the clean data were used to search against Rfam using the program cmsearch. Their default alignment parameters are as follows: Bowtie2: -q -L 16 --phred64 -p 6 cmsearch: --cpu 6 --noali. In the annotation information of different RNAs, some small RNA tags may be mapped to more than one category. To make sure each unique small RNA is mapped to only one category, we follow the priority rule: MiRbase> pirnabank> snoRNA (plant) > Rfam > other sRNA. We use miRA (for plants) to predict novel miRNA by exploring the characteristic hairpin structure of miRNA precursor. In order to find more accurate targets, multiple types of software are used. Generally, we use psRobot, TAPIR or Target Finder to predict plant targets. The default parameters are as follows: psRobot: -gl 17 -p 8 -gn 1, TargetFinder: -c 4, and TAPIR: --score 5 --mfe_ratio 0.6.

Desired sequences were small RNAs with no more than two mismatches, which were then aligned against ginger RNA sequences. Their flanking sequences were used to predict secondary structures with the assistance of MFOLD software. Conserved miRNAs were identified following the rule of the filtered small RNA containing a perfect stem-loop structure. miRDeep2 software was used to identify novel miRNAs and confirm the secondary structures of putative pre-miRNAs with modified parameters. The equation: MFEI = AMFE/(G + C)% was used to calculate the minimum free energy index (MFEI). The adjusted minimal folding free energy (AMFE), representing the MFE of 100 nucleotides, was calculated using the RNAfold webserver (http://rna.tbi.univie.ac.at/cgi-bin/RNAWebSuite/ RNAfold.cgi). All the obtained RNA sequences were used for target prediction using the TargetFinder and psRNA Target programs.

### Quantitative real-time PCR analysis of miRNA target genes

To validate the miRNAs from deep sequencing, the expression patterns were analyzed using the stem-loop RT-PCR. Following the manufacturer’s instruction, the total RNA of ginger rhizome in three development stages was extracted using TRIzol reagent (Invitrogen). Then, the total RNA was reverse-transcribed to generate cDNA using the stem-loop RT primer and the PrimeScript RT reagent kit (Takara, Dalian, China) following the manufacturer’s protocol. All the primers listed in Table S5 for stem-loop qRT-PCR were designed according to Xu et al. [41].

The qRT-PCR reactions were performed using the following conditions: 95 °C for 5 min, 40 cycles of denaturation consisting of 15 s at 95 °C, 30 s at 60 °C, and 30 s at 72 °C, using SYBR Premix Ex Taq II solution (TaKaRa, Dalian, China). For the qRT-PCR analysis of the miRNA targets, total RNA was used as a template to generate reverse transcripts using the PrimeScript RT reagent kit following the manufacturer’s instructions. For each miRNA target, specific primer pairs were designed to amplify the cDNA (Supplementary Table S5). qPCR was performed using SYBR Premix Ex Taq II using the conditions of 95 °C for 15 s with 40 cycles consisting of 60 °C for 30 s, and 72 °C for 30 s. Each sample was processed in triplicate, and the 2^−ΔΔCT^ method was used to calculate the relative expression levels [42]. To normalize the expression level of miRNAs and their targets, the *RBP* genes were used as references. All primers used in this study are shown in Supplementary Table S5. SAS Version 9.0 (SAS Institute, Cary, NC, USA) software was used to analyze the data using Duncan’s multiple range test. The level of significance was defined at *P* < 0.05.

## Supplementary Information

**Additional file 1: Supplementary Table S1.** Statistics of sequencing reads from leaf, root, rhizome, stem and flower libraries of *Z. officinale* Roscoe.

**Additional file 2: Supplementary Table S2.** Conserved miRNAs identified from the flower, leaf, root, stem, and rhizome libraries of *Z. officinale* Roscoe.

**Additional file 3: Supplementary Table S3.** Novel miRNAs identified from the flower, leaf, root, stem, and rhizome libraries of *Z. officinale* Roscoe.

**Additional file 4: Supplementary Figure S1.** The stem-loop structures of conserved *Z. officinale* Roscoe miRNA precursors.

**Additional file 5: Supplementary Figure S2.** Expression profile of conserved miRNAs during rhizome development. Rz_1: Rhizome 1st I_d; Rz_2: Rhizome 2nd I_d; Rz_3: Rhizome 3rd I_d.

**Additional file 6: Supplementary Figure S3.** Expression profiles of novel miRNAs during rhizome development. Rz_1: Rhizome 1st I_d; Rz_2: Rhizome 2nd I_d; Rz_3: Rhizome 3rd I_d.

**Additional file 7: Supplementary Table S4.** Conserved and novel miRNA targets involved in rhizome development of *Z. officinale* Roscoe.

**Additional file 8: Supplementary Table S5.** All the primers used in this study.

## Data Availability

All data that support the findings of this study are available from the corresponding author upon reasonable request.
