# Supplementary material for: Genome-wide investigation of microRNAs and expression profiles during rhizome development in ginger (Zingiber officinale Roscoe)
Source: BMC Genomics. 2022 Jan 13;23:49. doi: 10.1186/s12864-021-08273-y (PMC8756691; doi:10.1186/s12864-021-08273-y)
Supplement: Supplementary file 4 — Additional file 4: Supplementary Figure S1. The stem-loop structures of conserved Z. officinale Roscoe miRNA precursors. [file 12864_2021_8273_MOESM4_ESM.pdf]

Supplementary Figure S1 The stem-loop structures of conserved *Z. officinale* Roscoe miRNA precursors.

novel_mir102

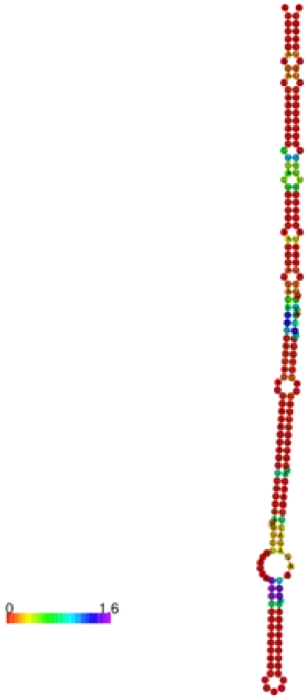

novel_mir103

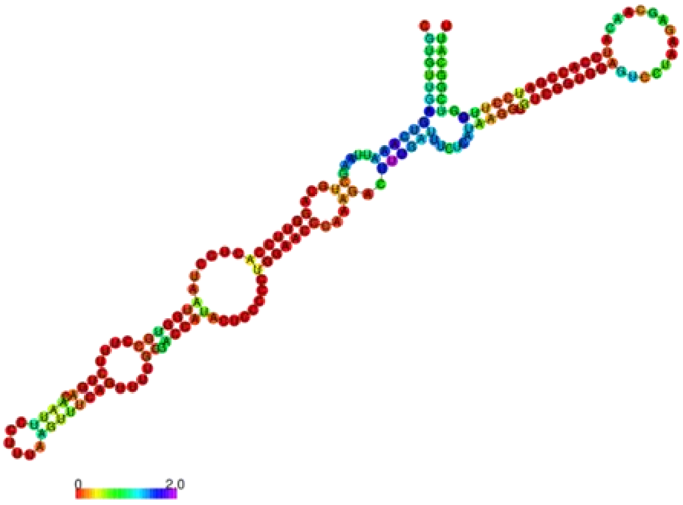

novel_mir104

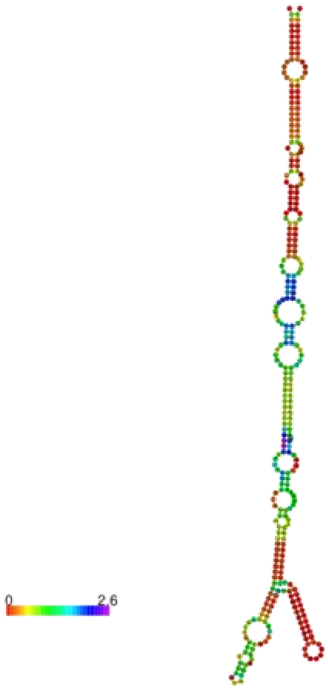

novel_mir105

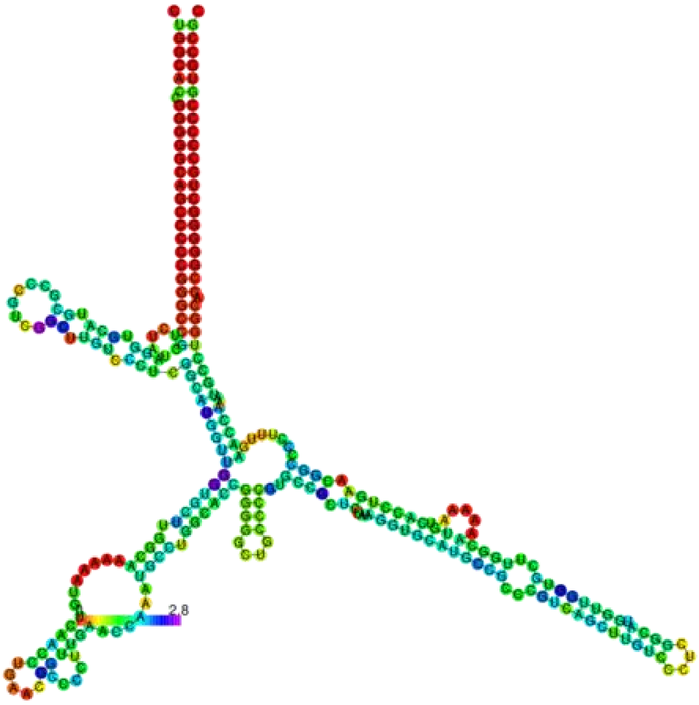

novel_mir106

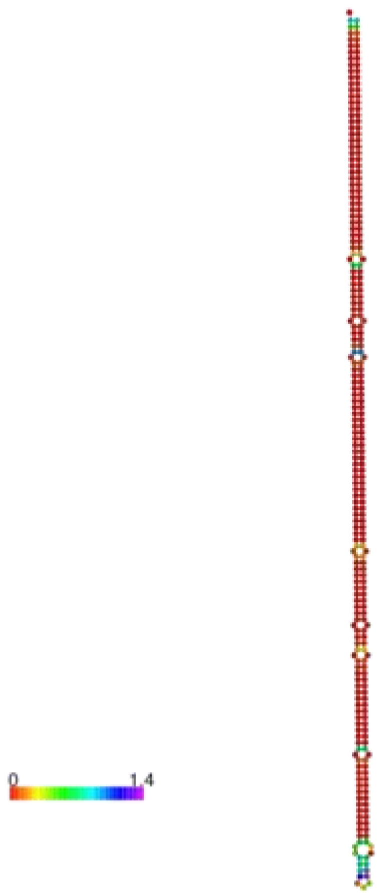

novel_mir108

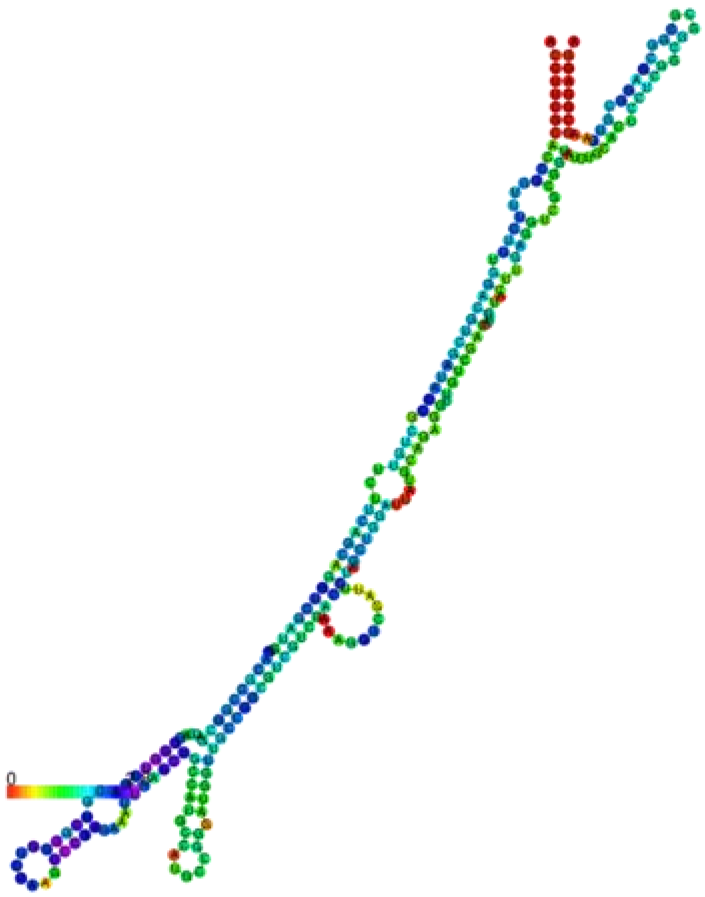

novel_mir109

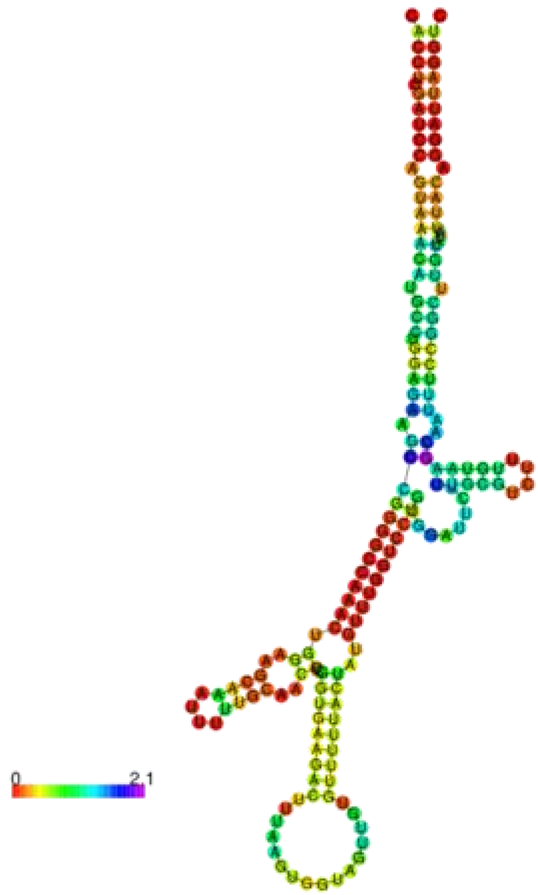

novel_mir11

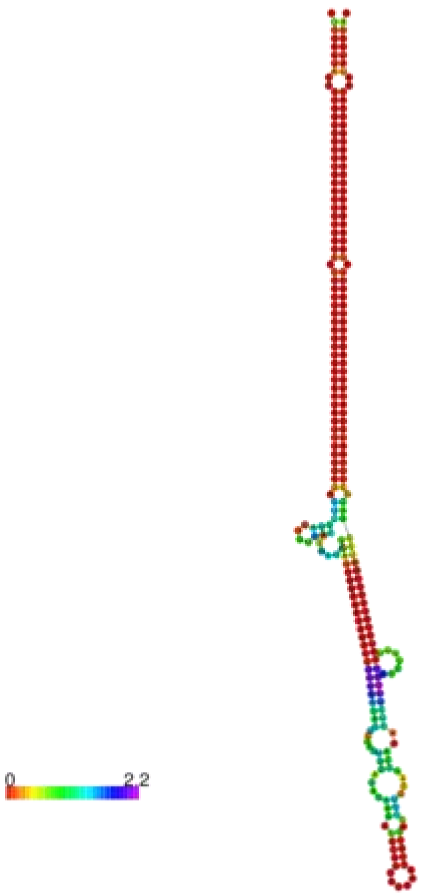

novel_mir110

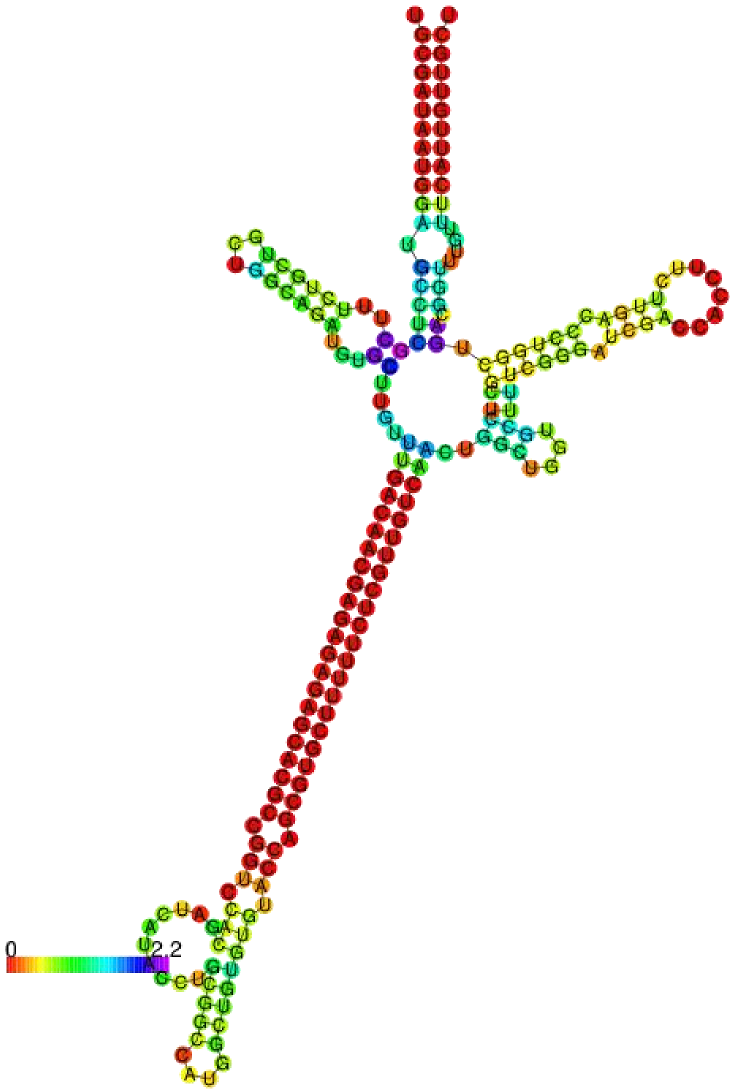

novel_mir111

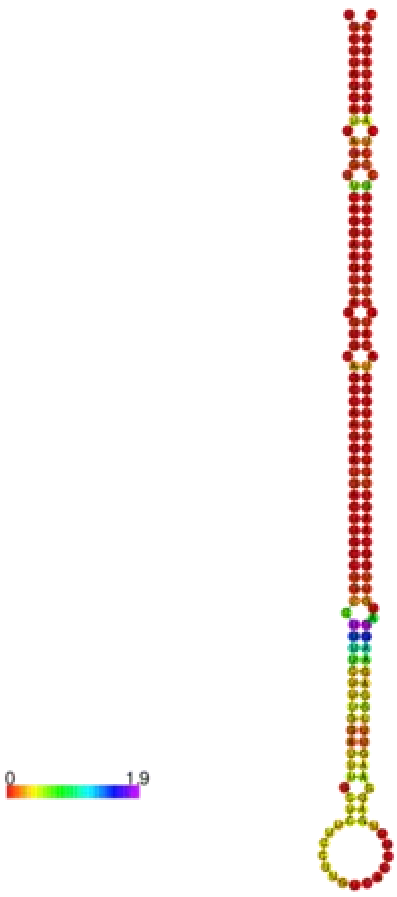

novel_mir112

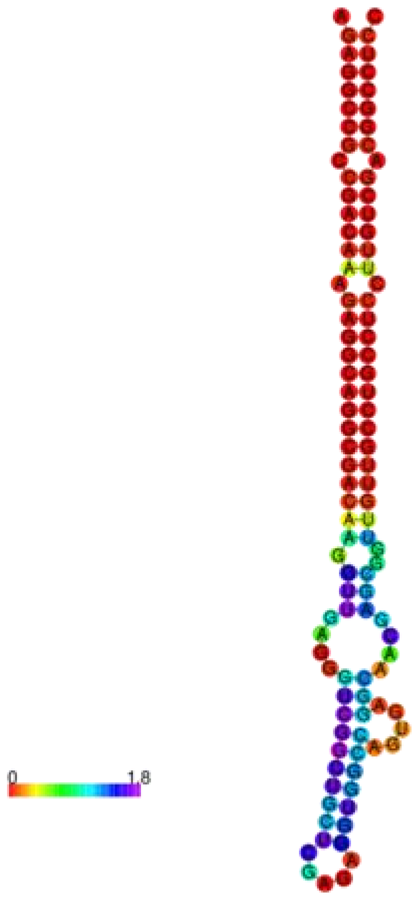

novel_mir115

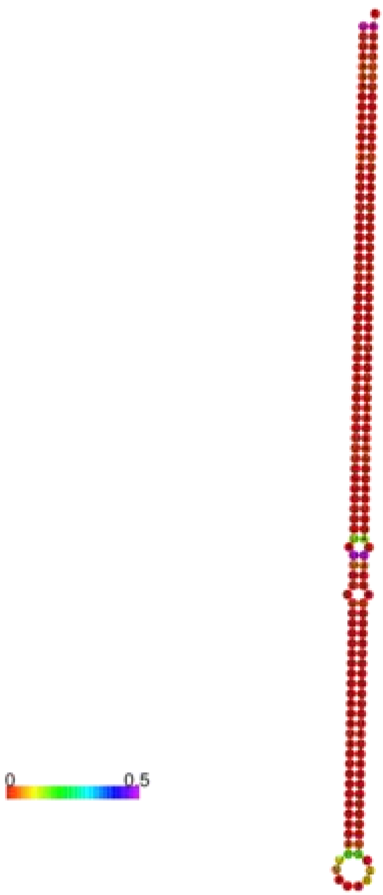

novel_mir117

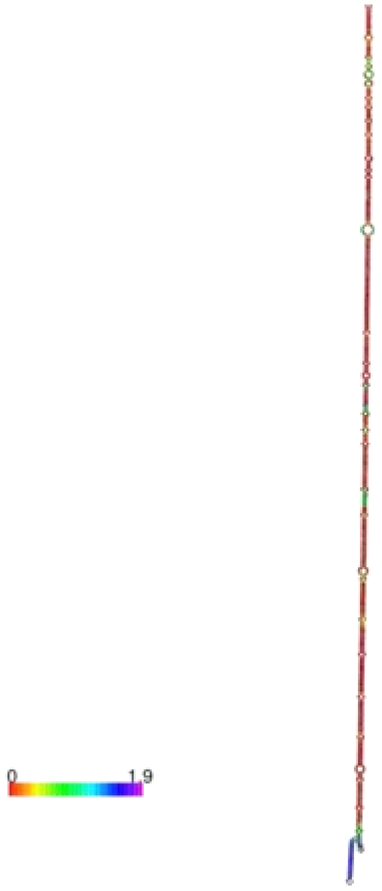

novel_mir120

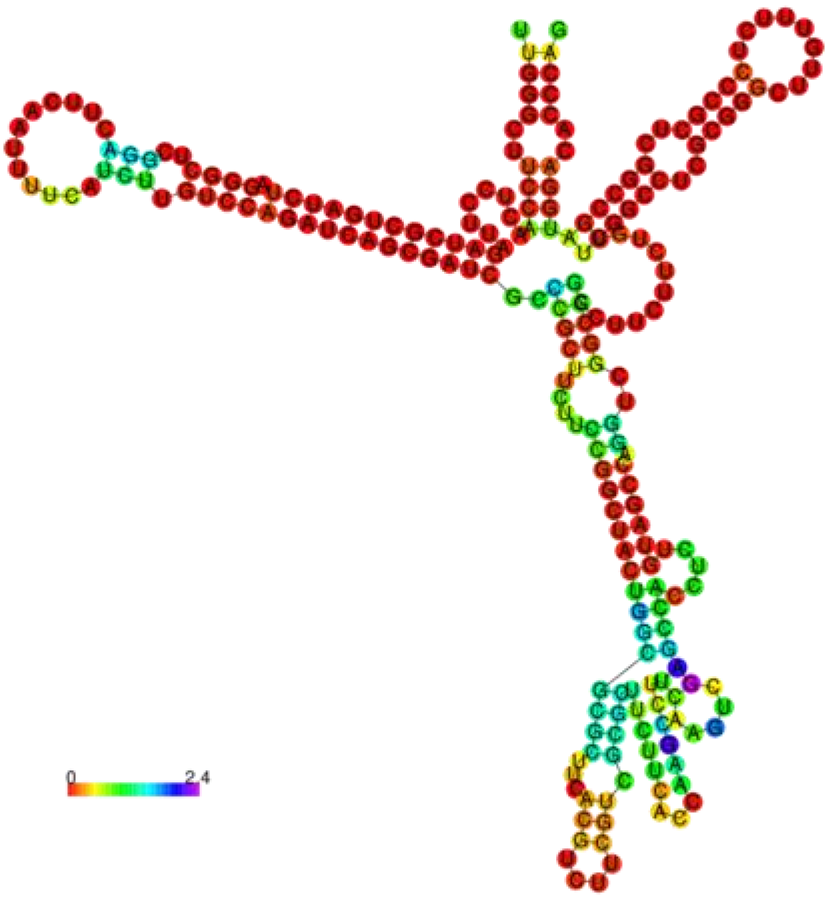

novel_mir121

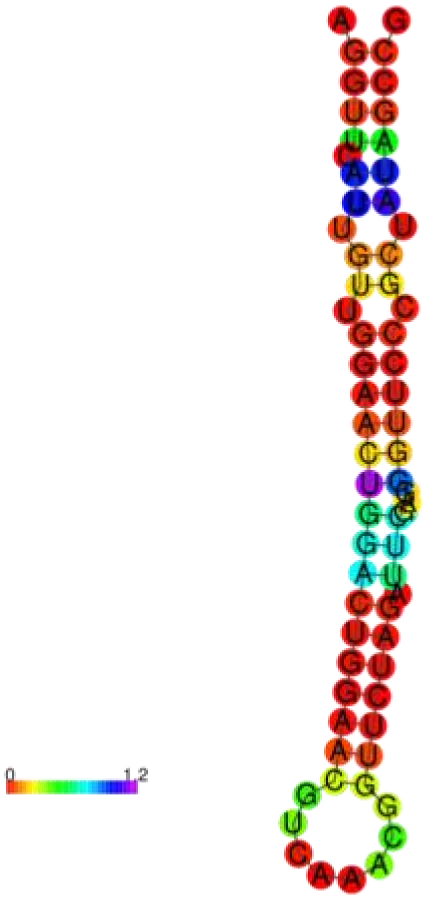

novel_mir122

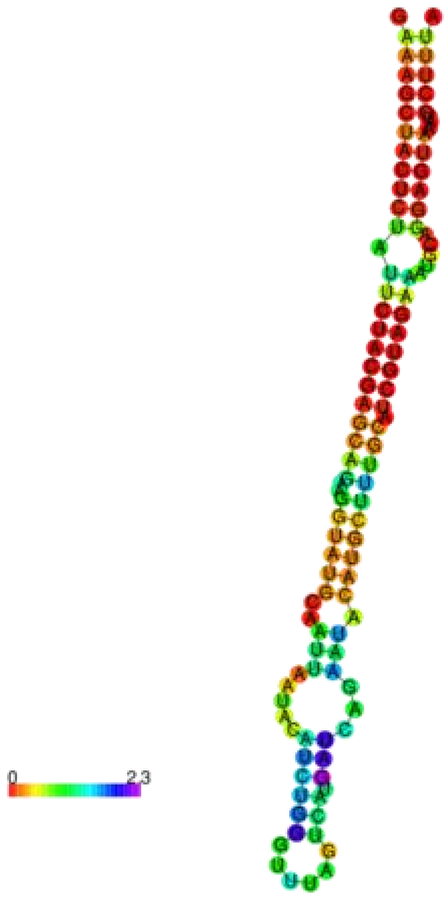

novel_mir125

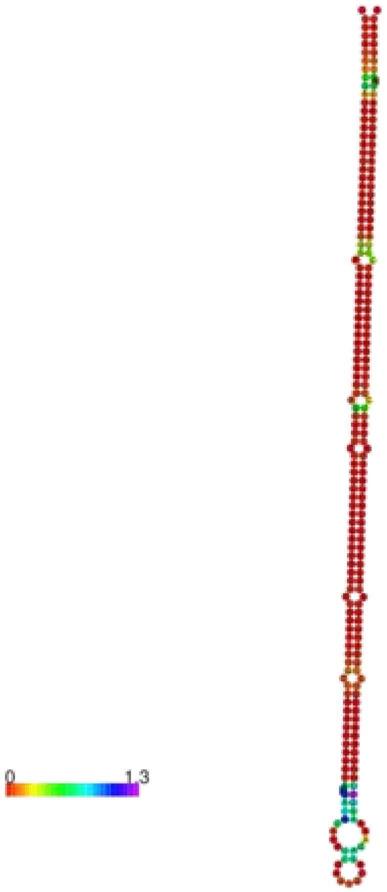

novel_mir126

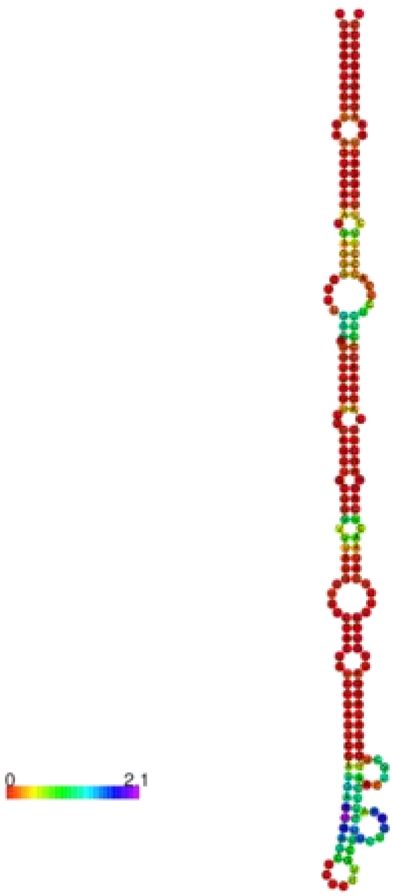

novel_mir127

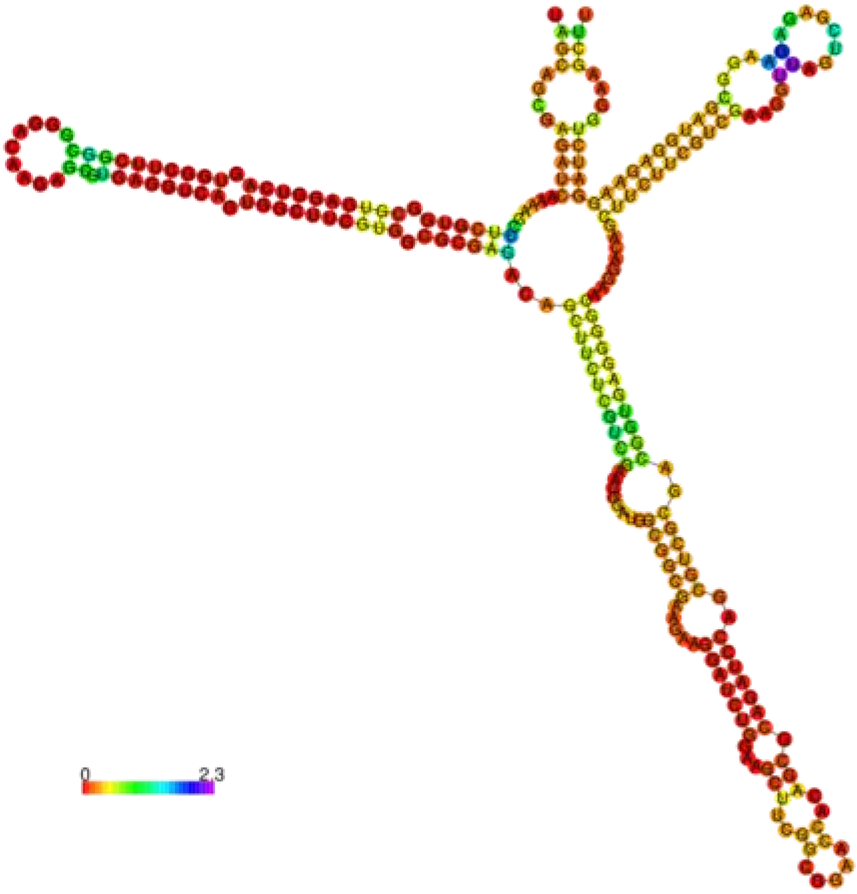

novel_mir129

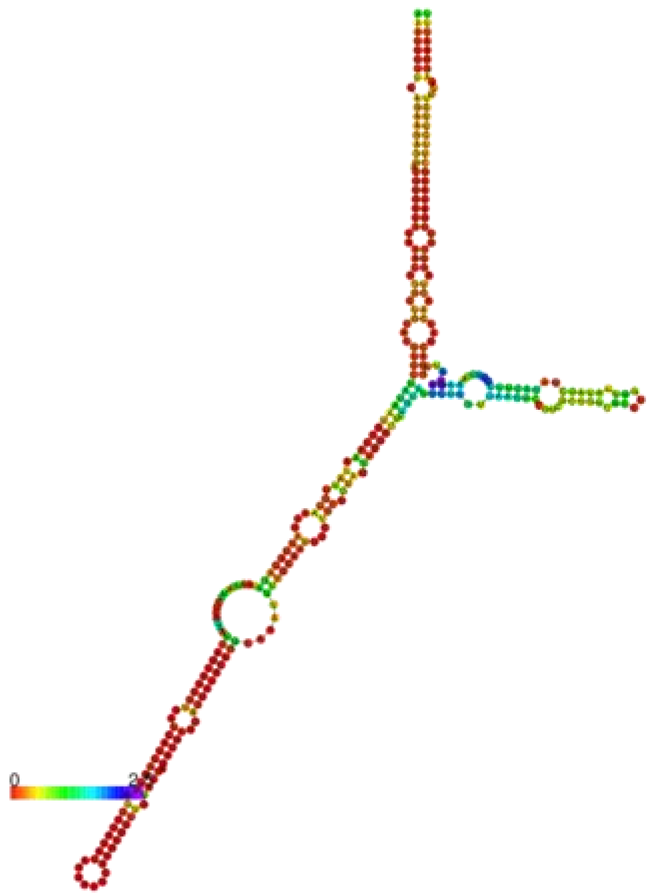

novel_mir13

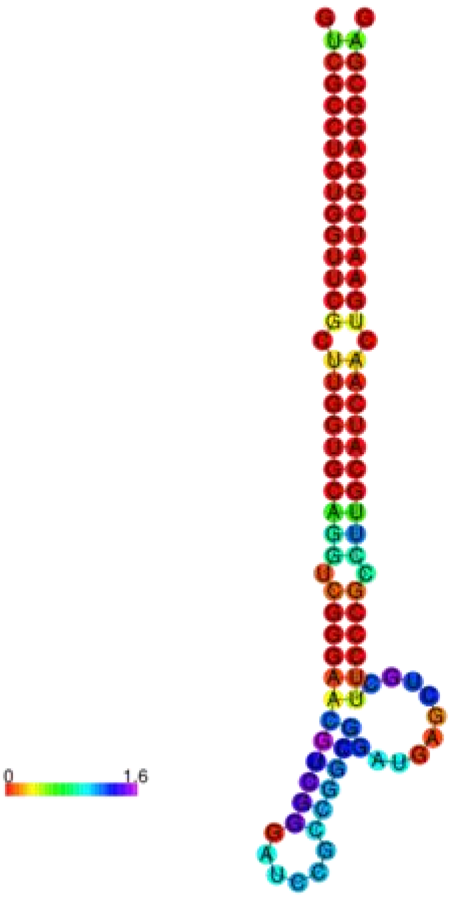

novel_mir130

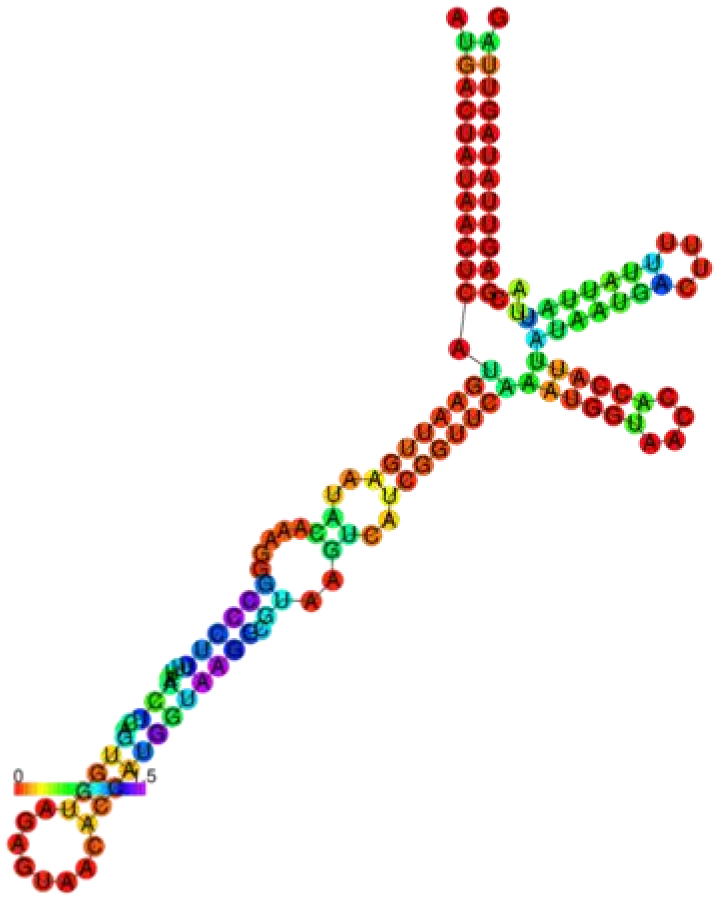

novel_mir133

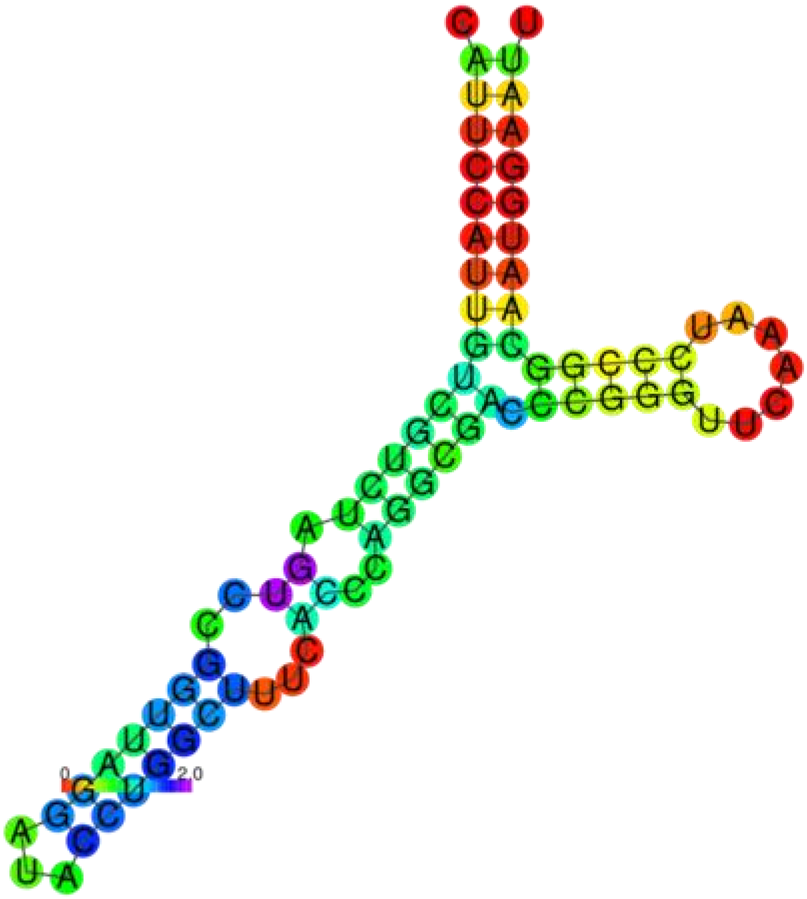

novel_mir134

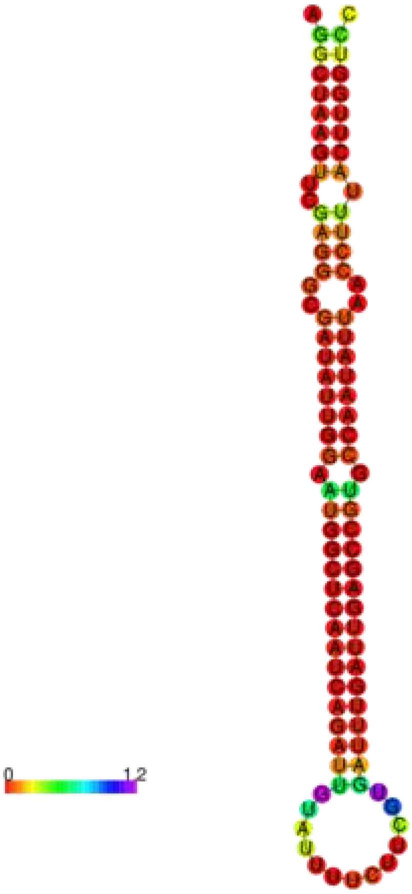

novel_mir135

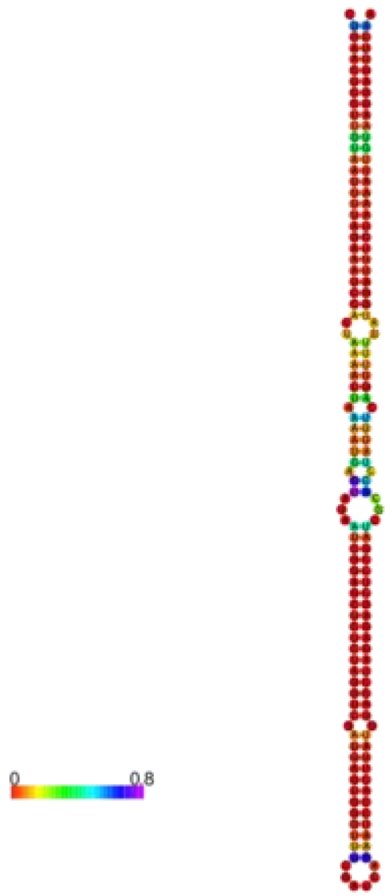

novel_mir136

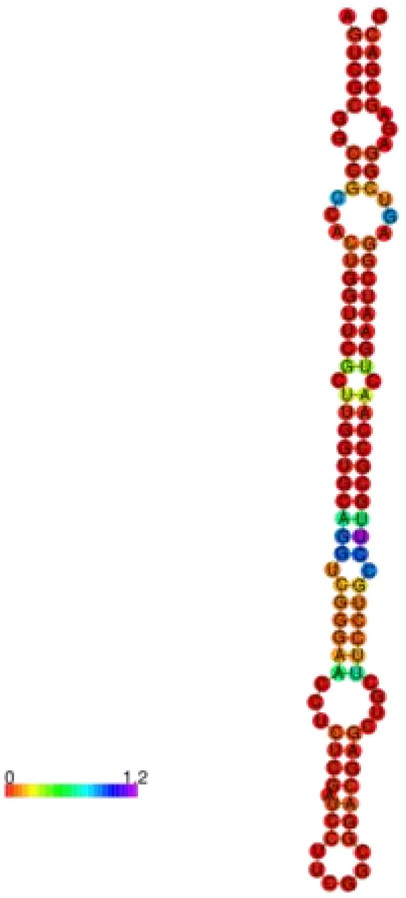

novel_mir137

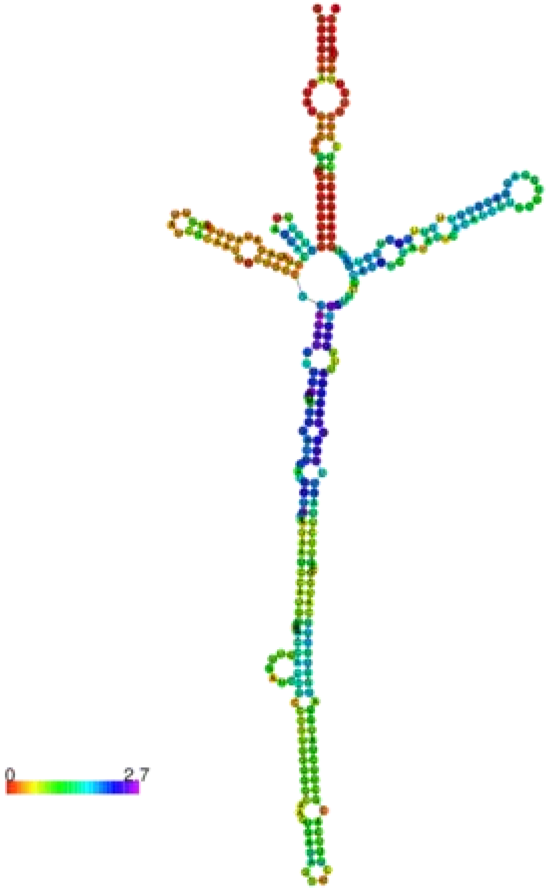

novel_mir139

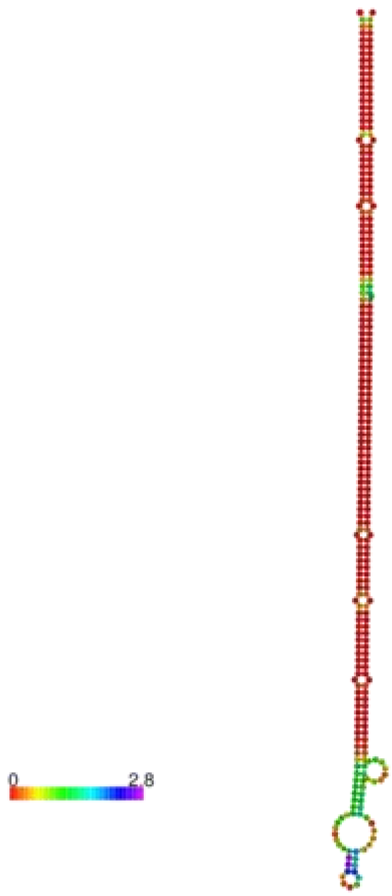

novel_mir14

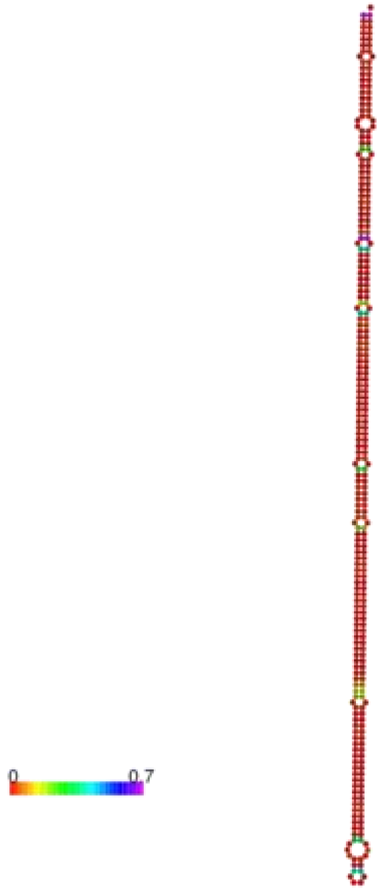

novel_mir140

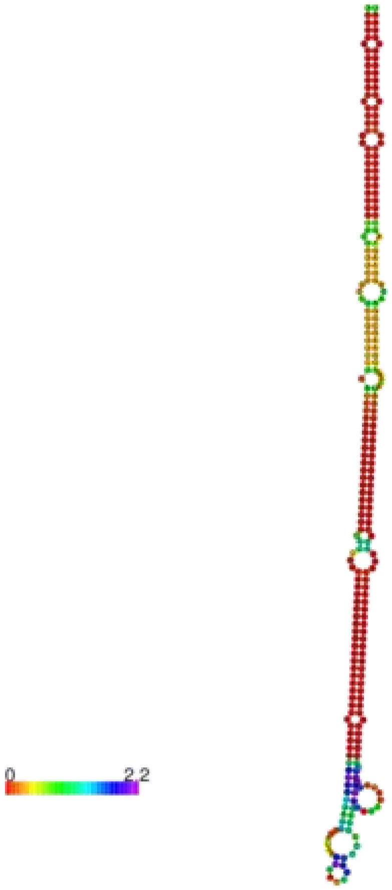

novel_mir142

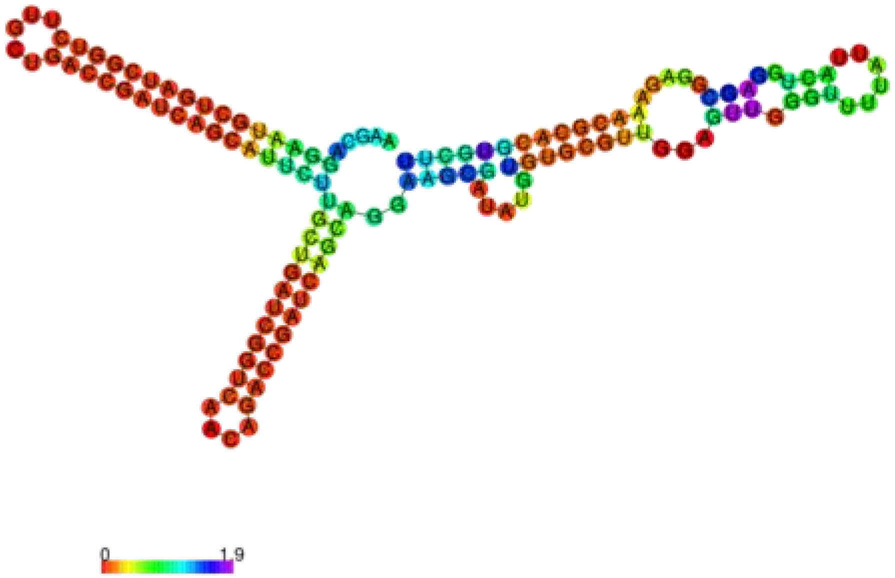

novel_mir148

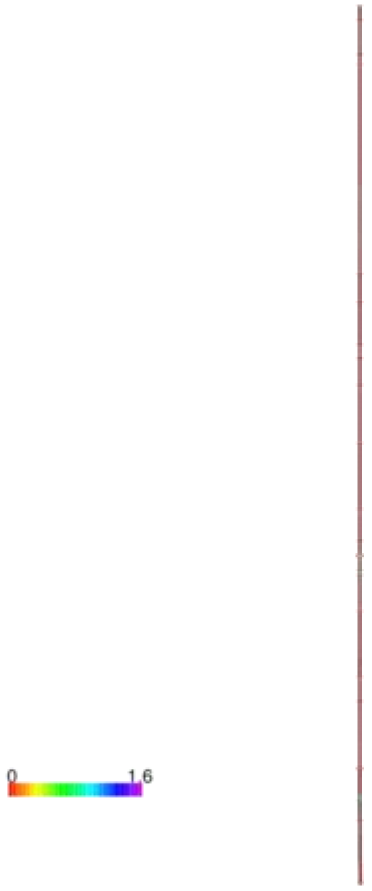

novel_mir149

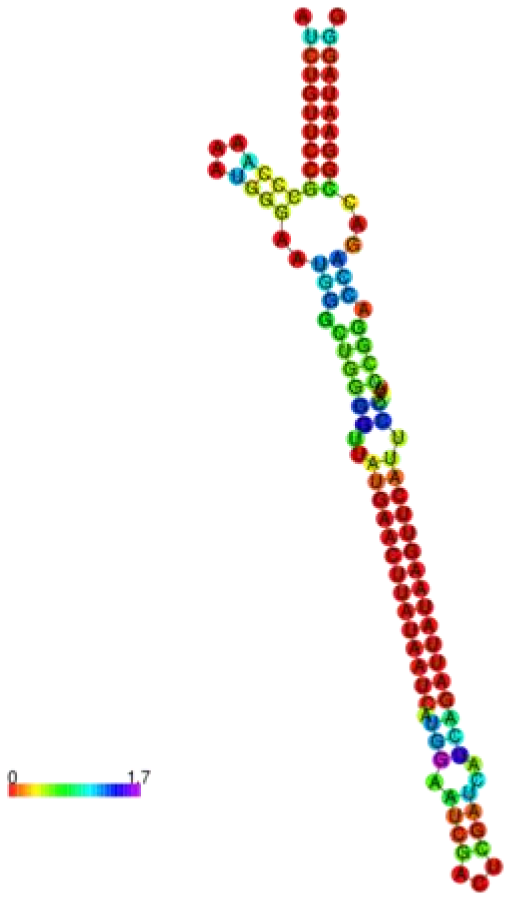

novel_mir151

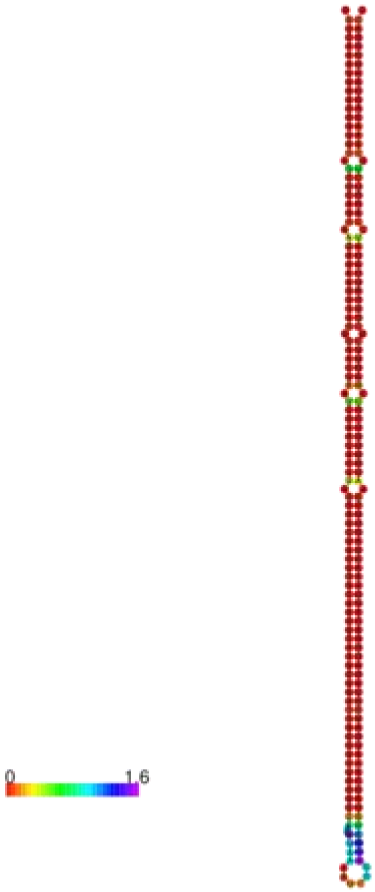

novel_mir153

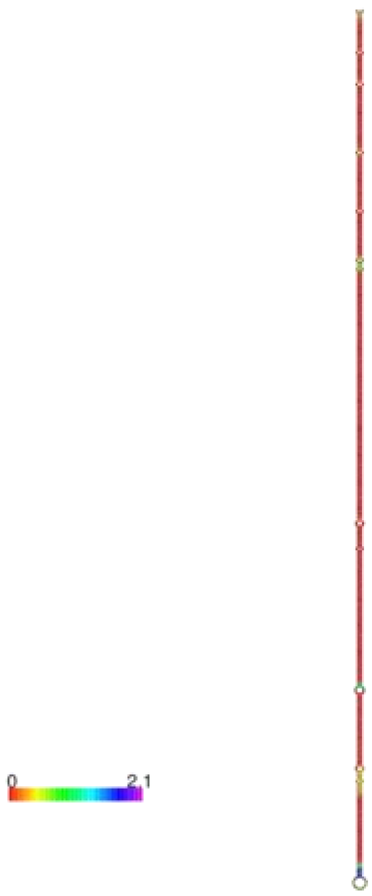

novel_mir154

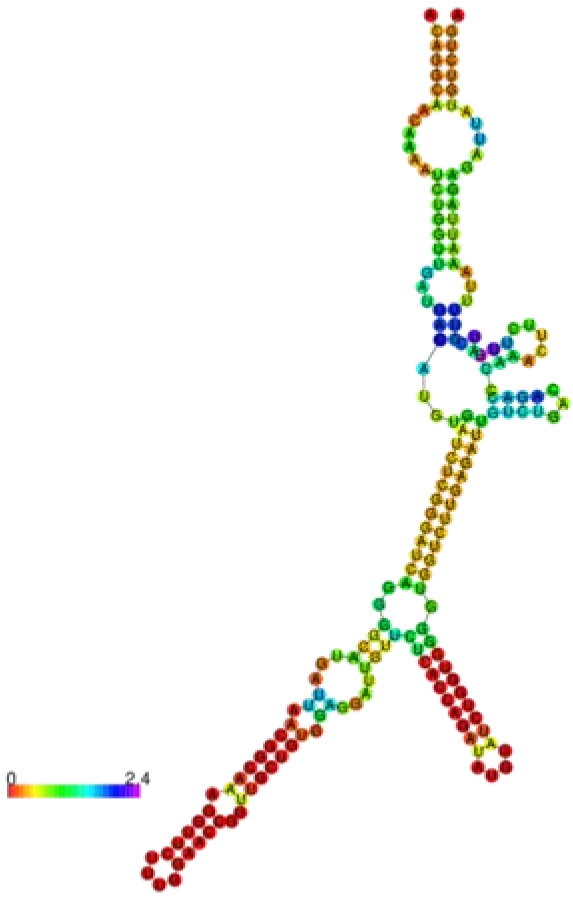

novel_mir155

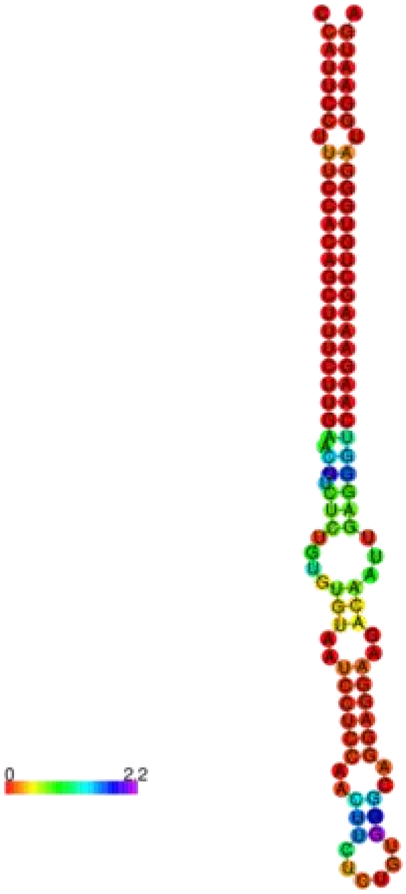

novel_mir157

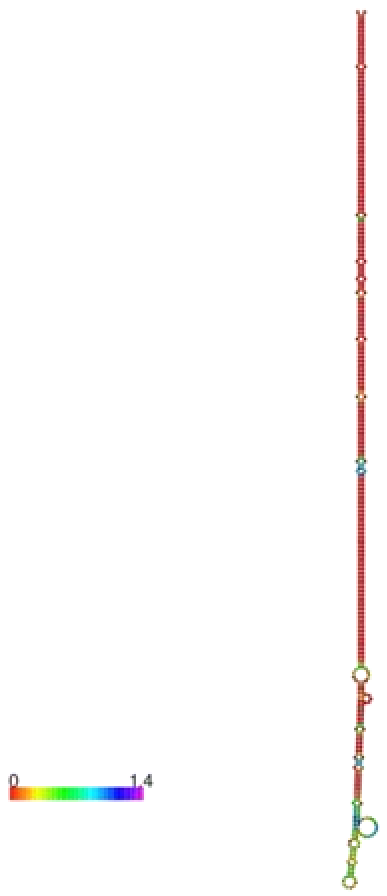

novel_mir158

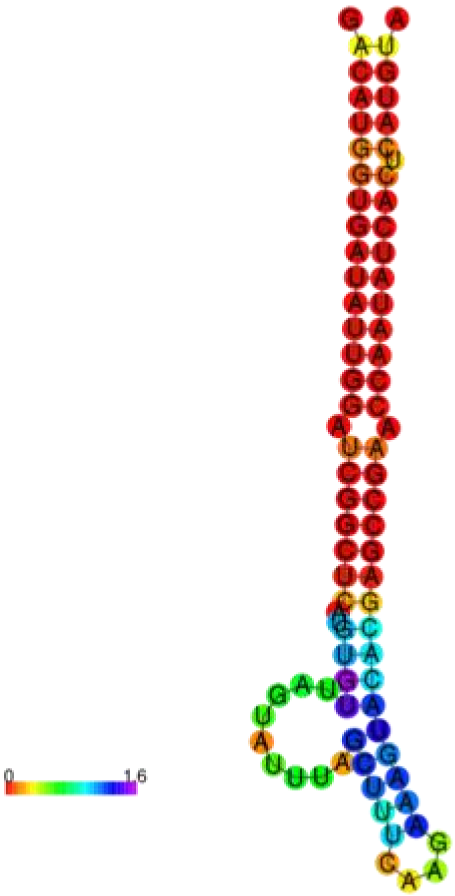

novel_mir159

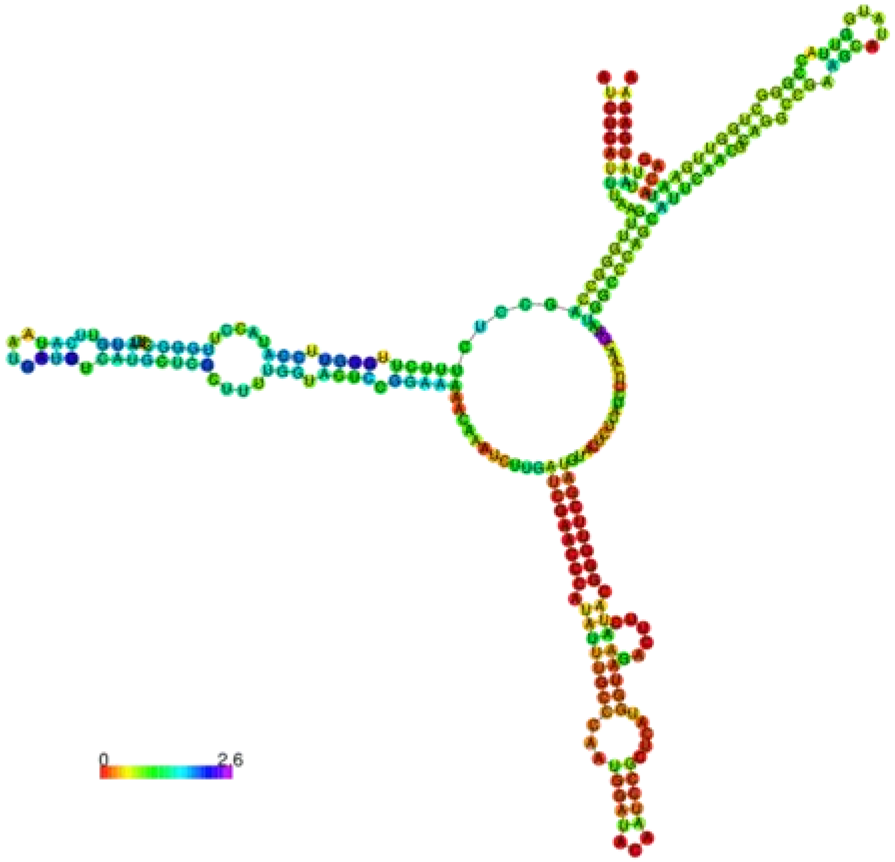

novel_mir166

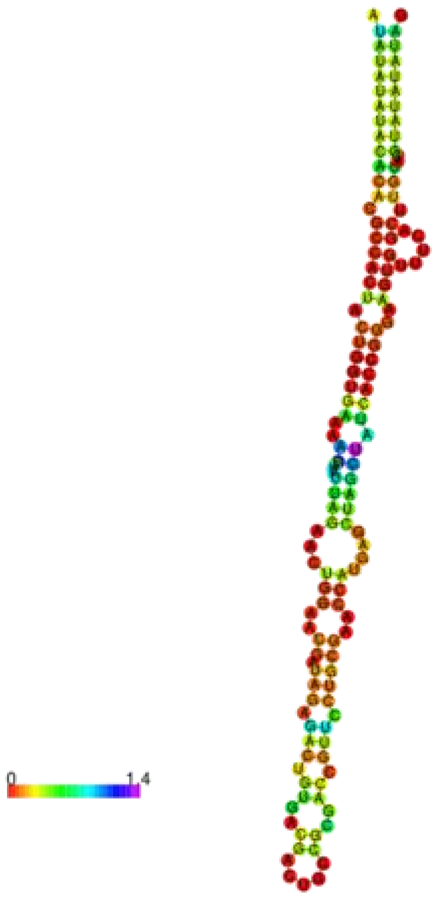

novel_mir168

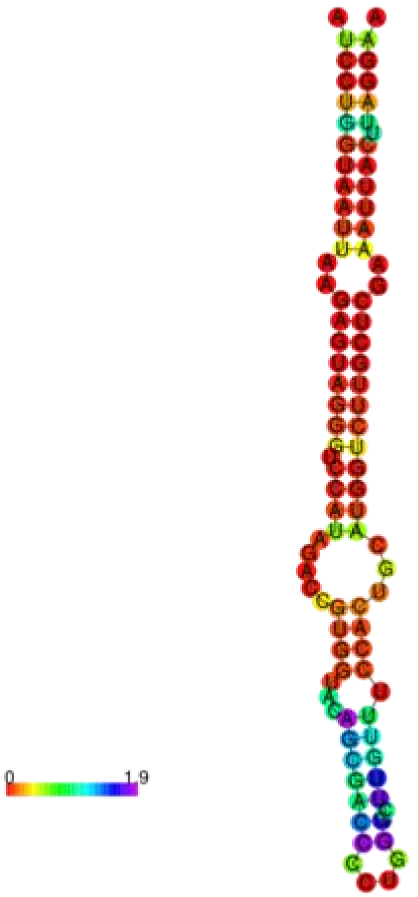

novel_mir169

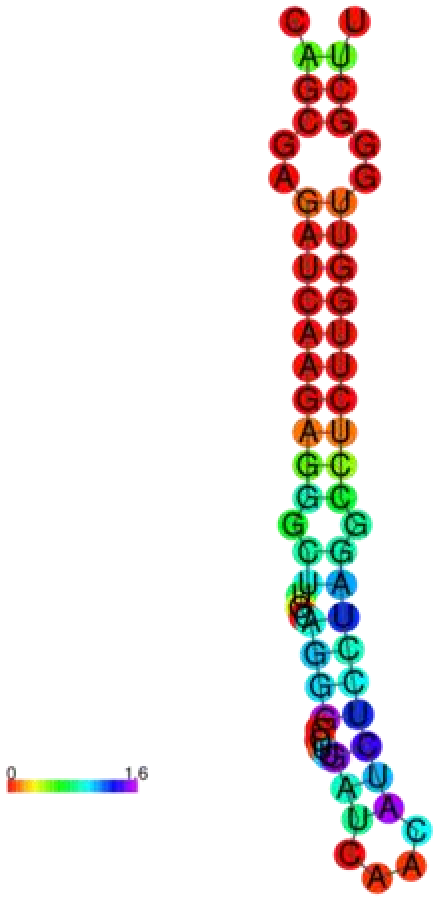

novel_mir17

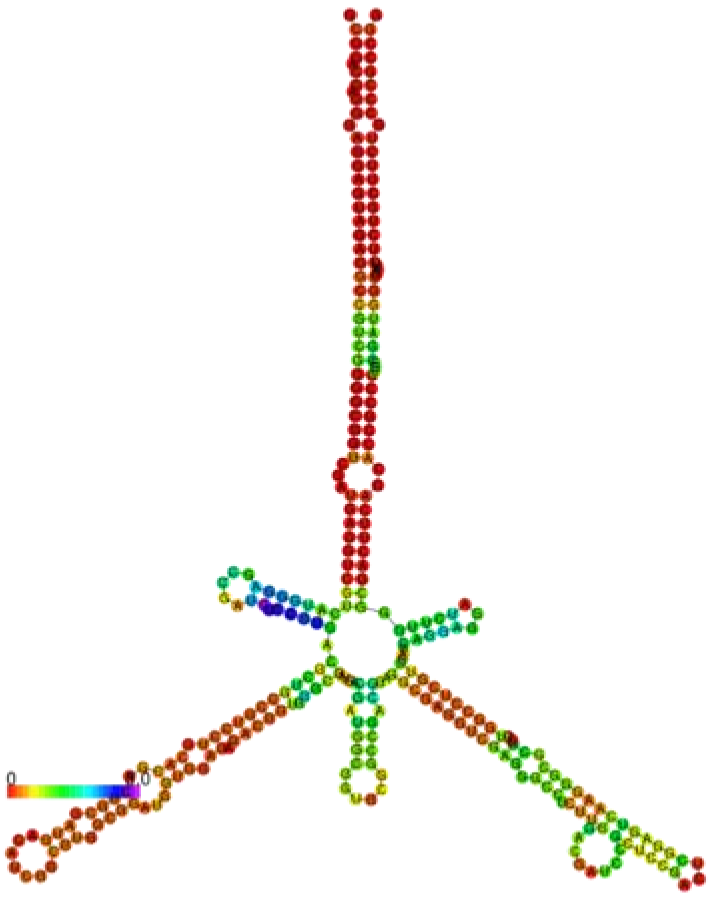

novel_mir171

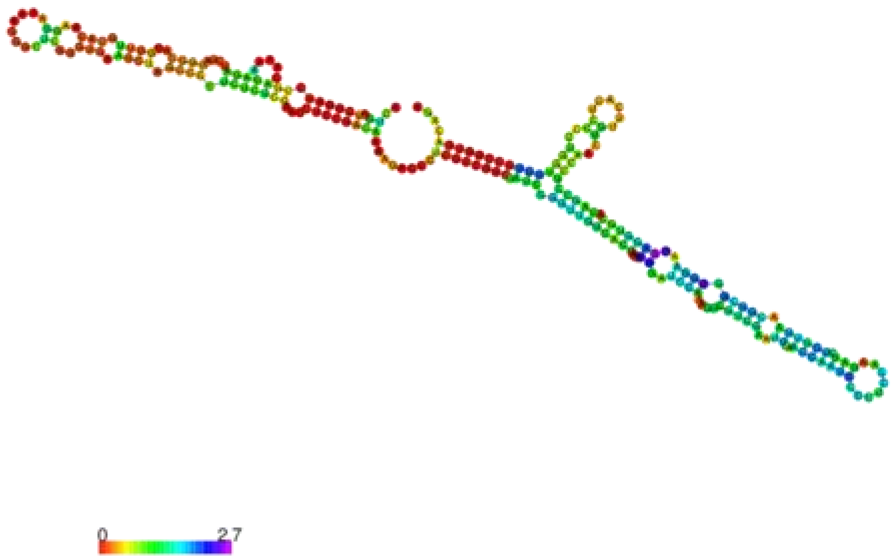

novel_mir174

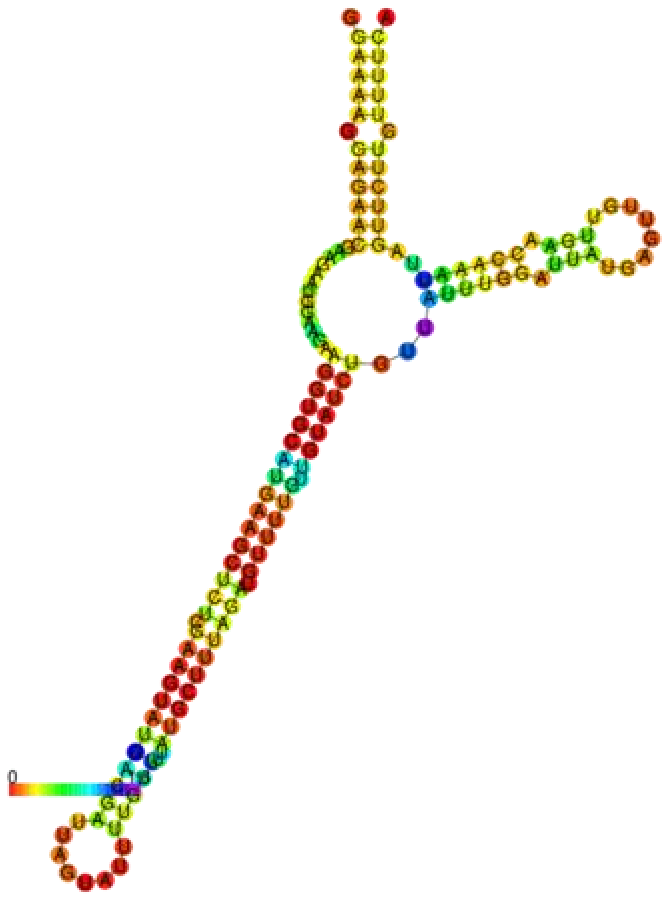

novel_mir177

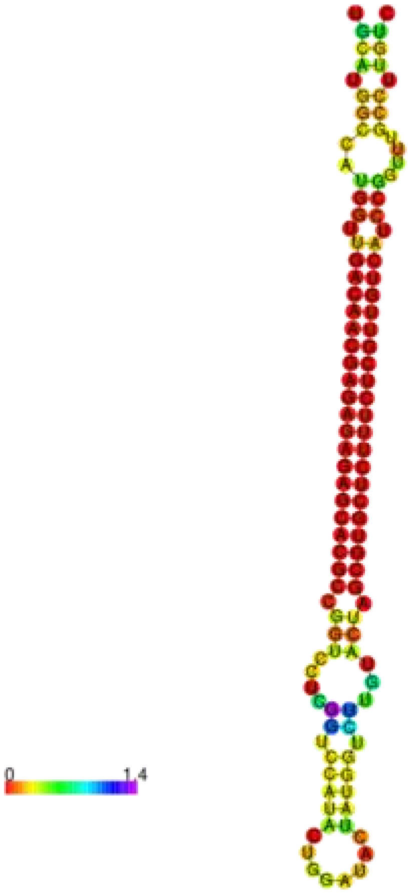

novel_mir179

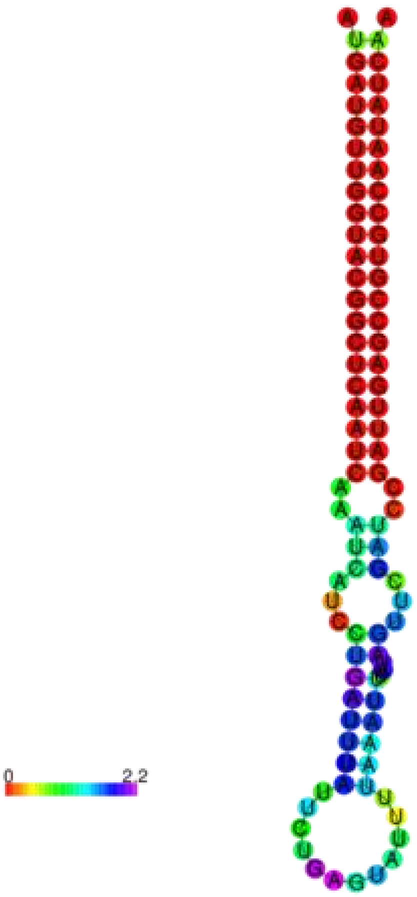

novel_mir18

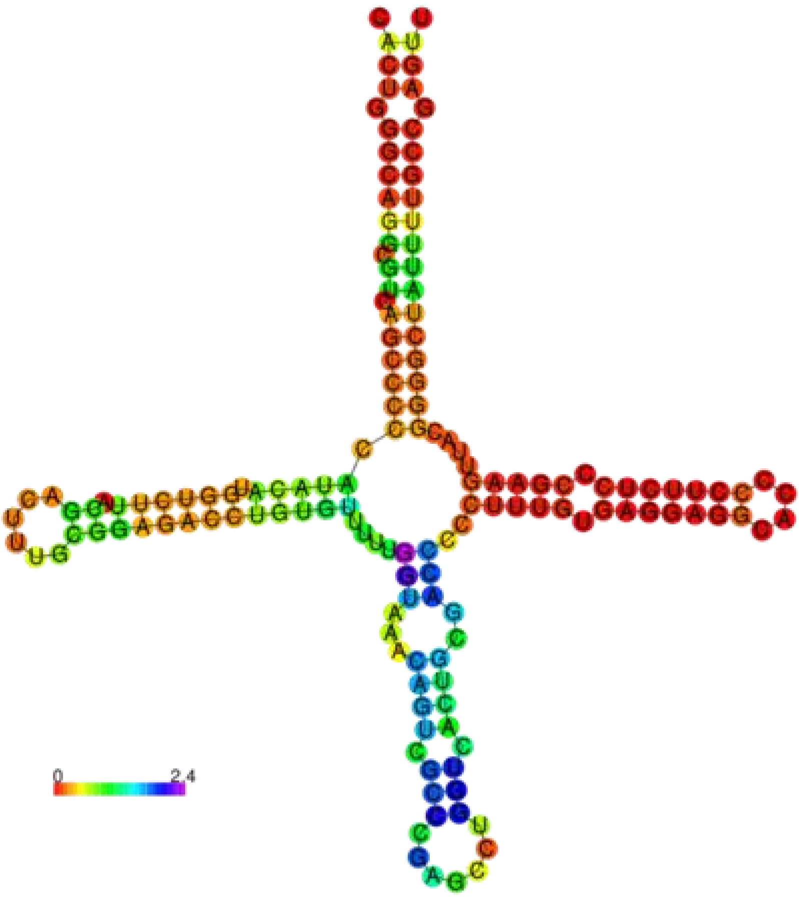

novel_mir181

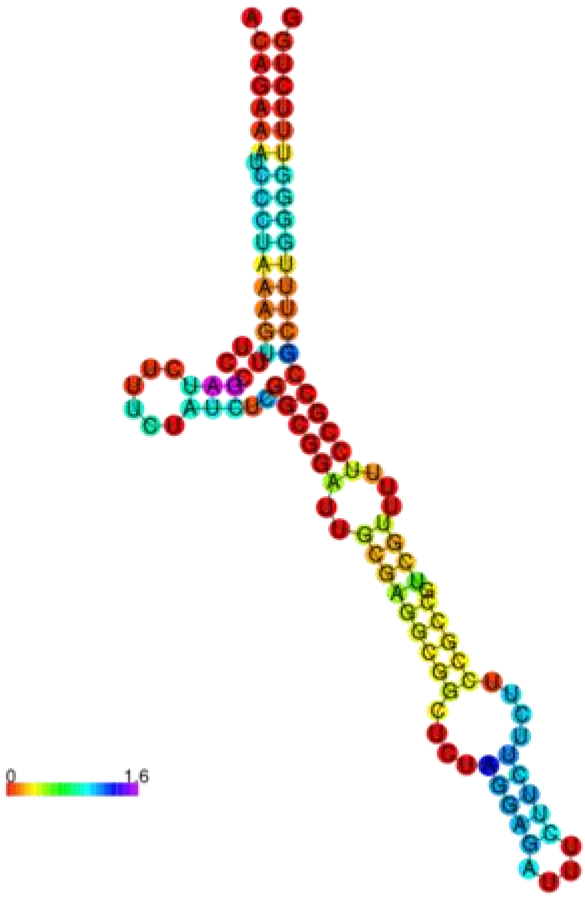

novel_mir187

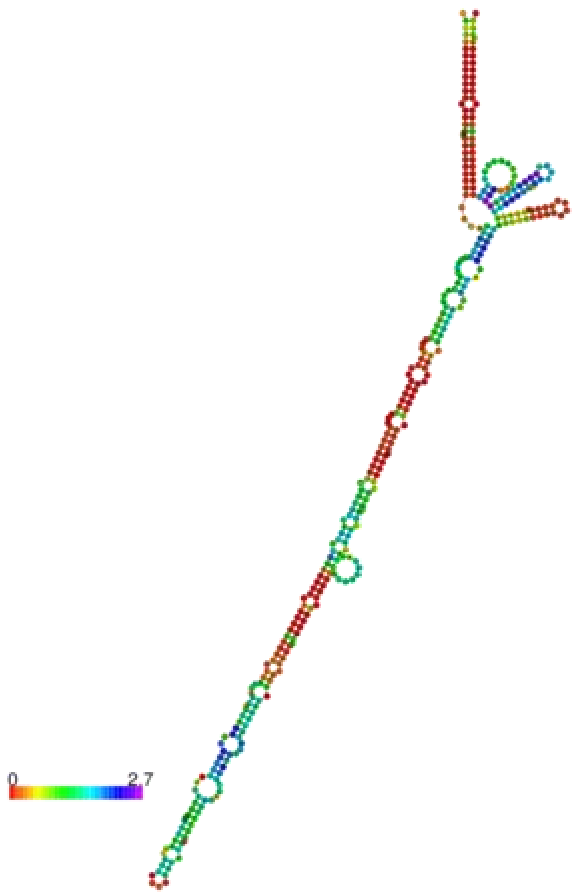

novel_mir188

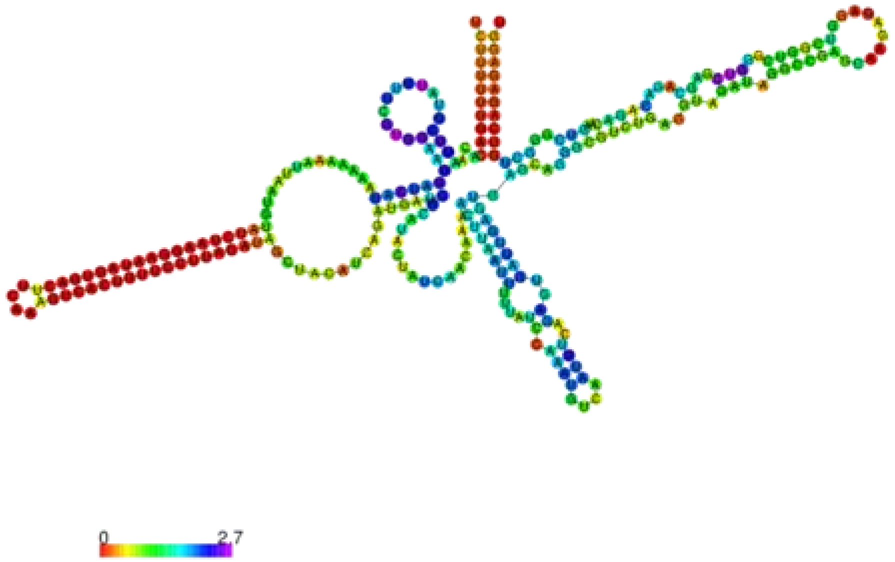

novel_mir19

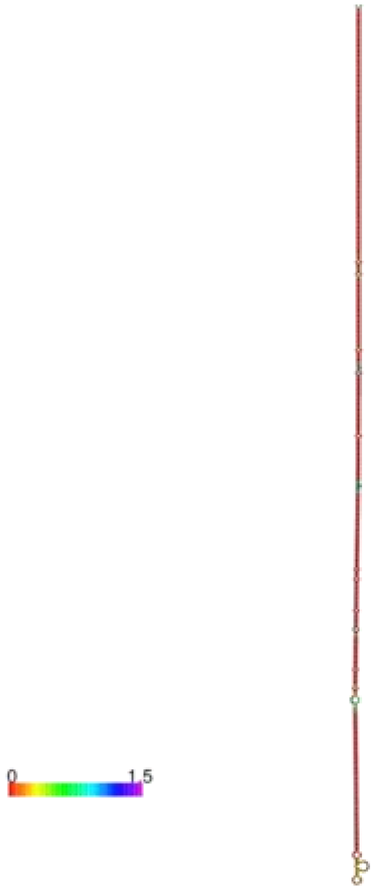

novel_mir192

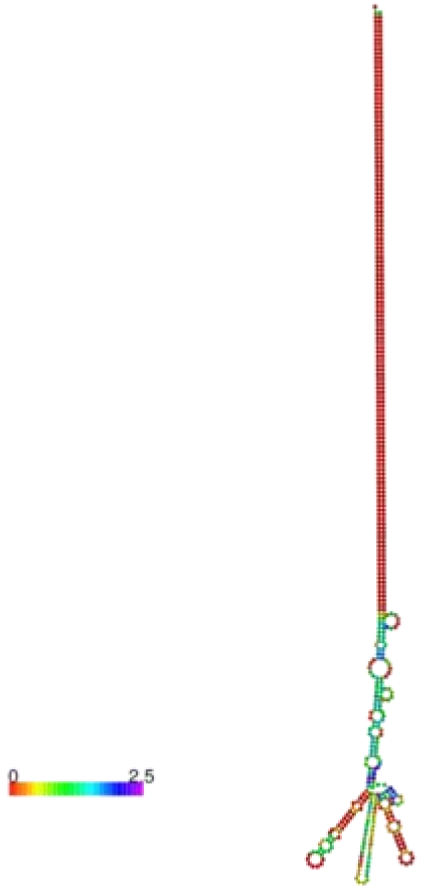

novel_mir196

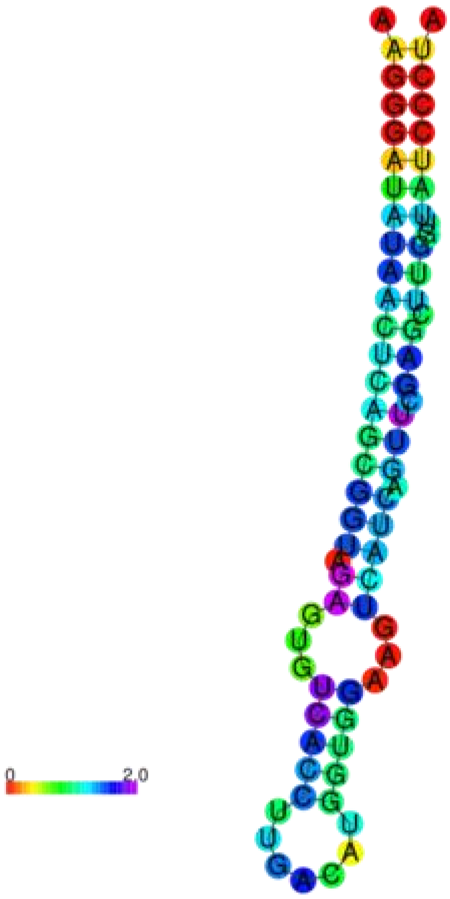

novel_mir201

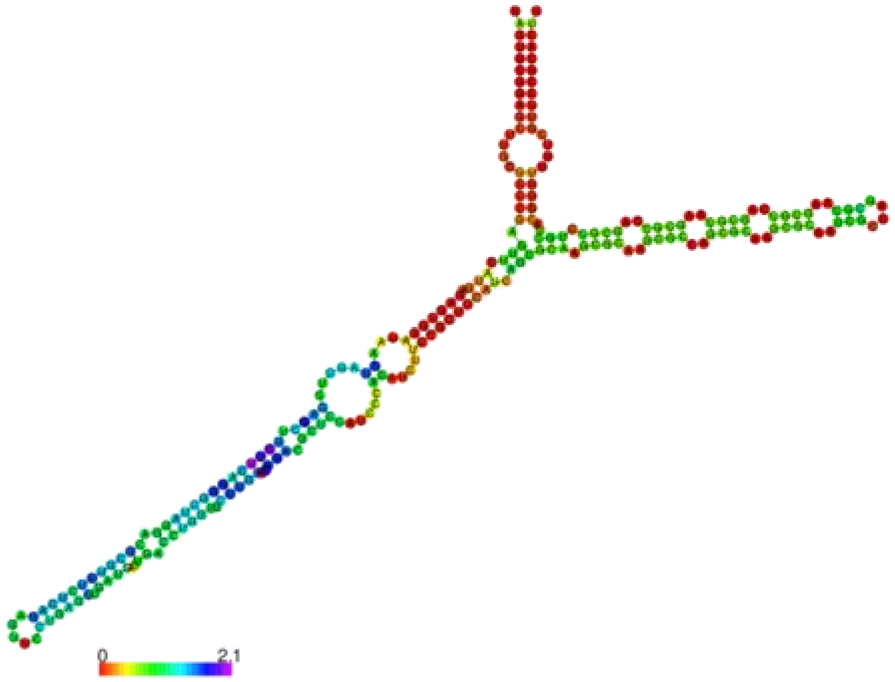

novel_mir205

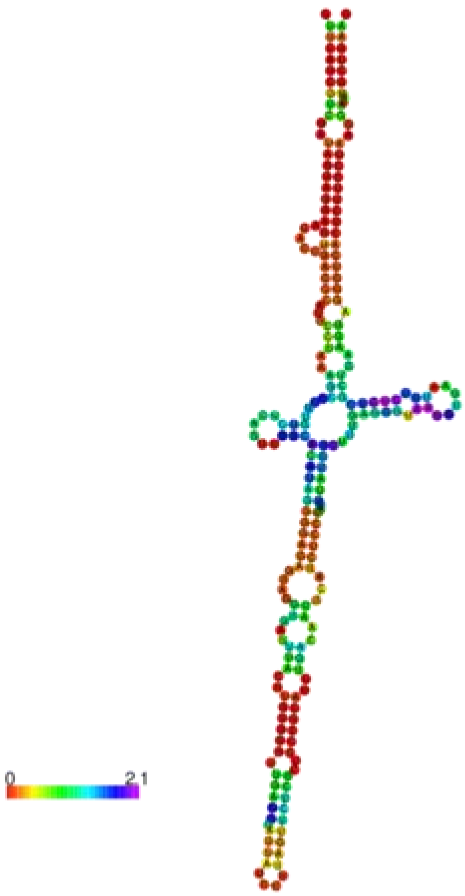

novel_mir207

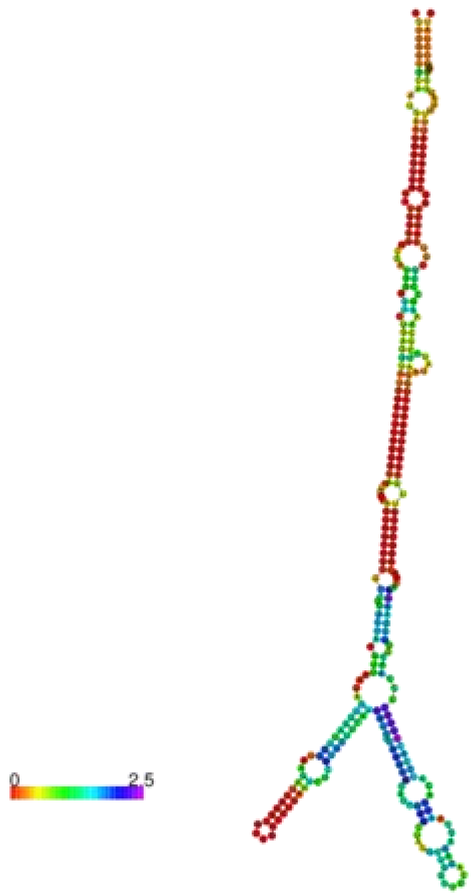

novel_mir209

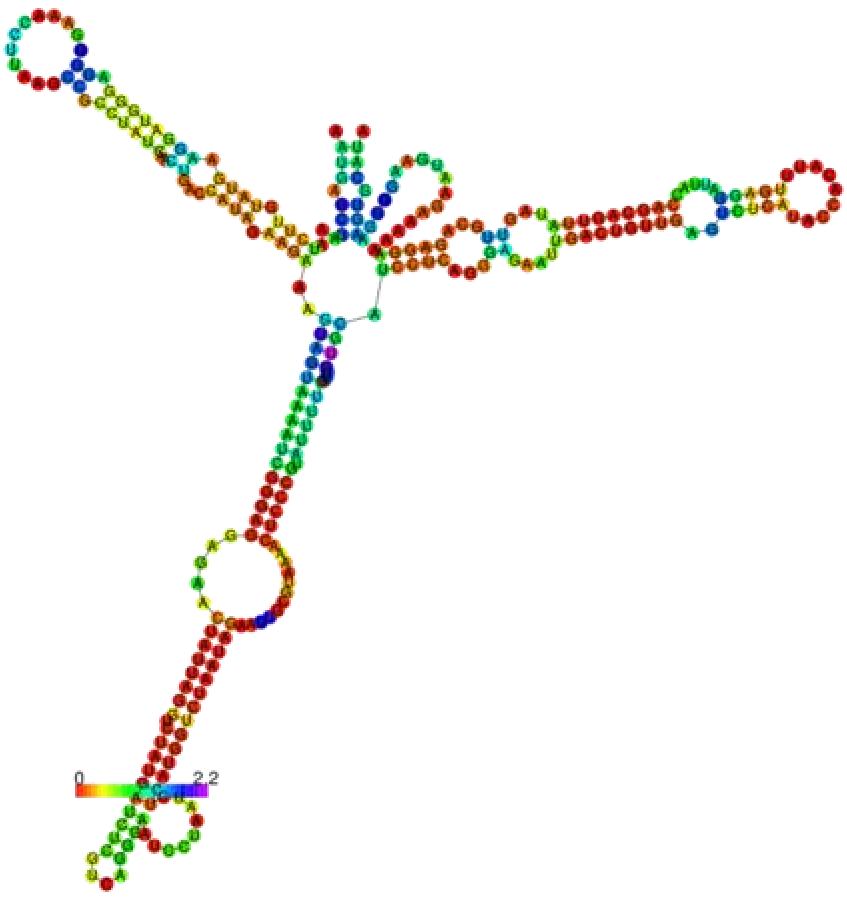

novel_mir21

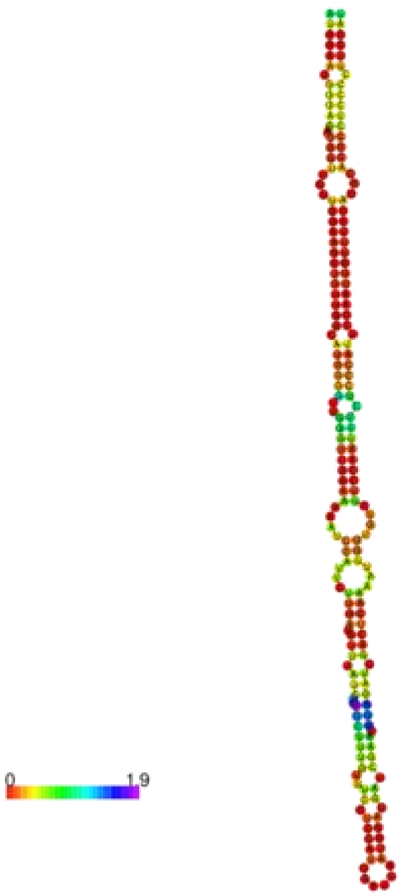

novel_mir212

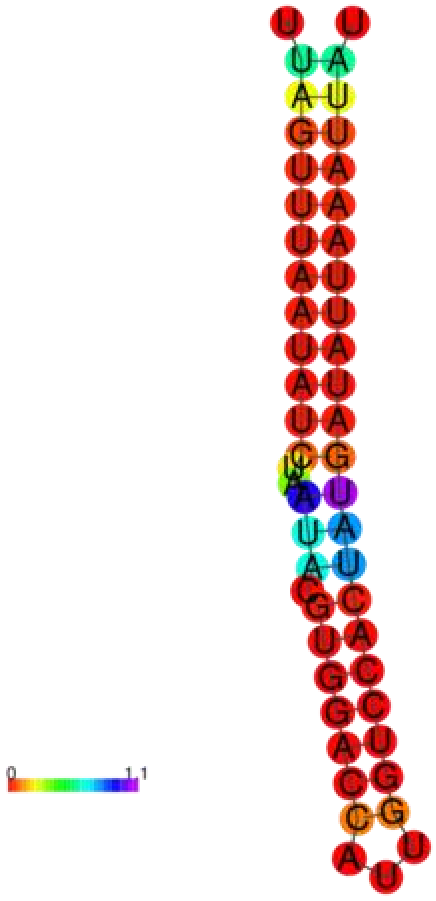

novel_mir213

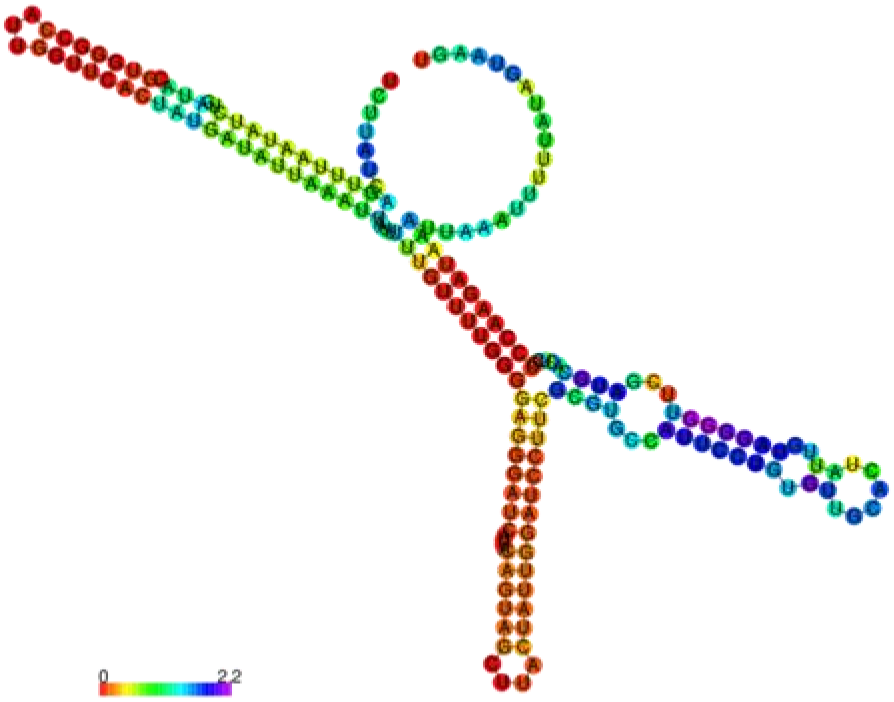

novel_mir214

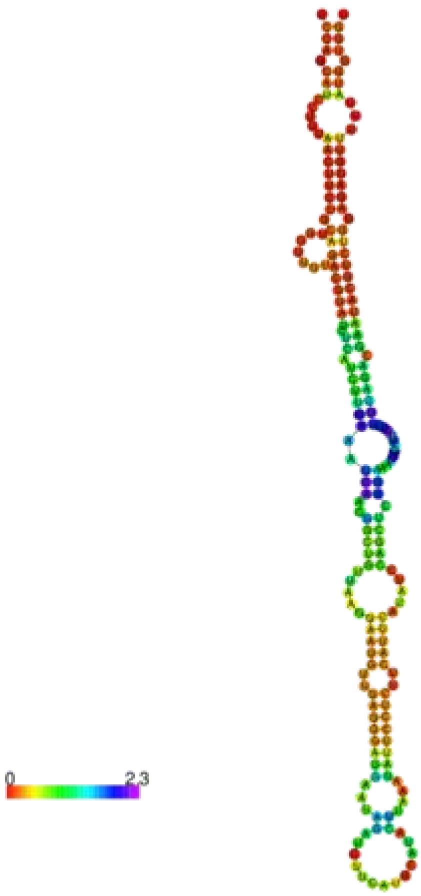

novel_mir22

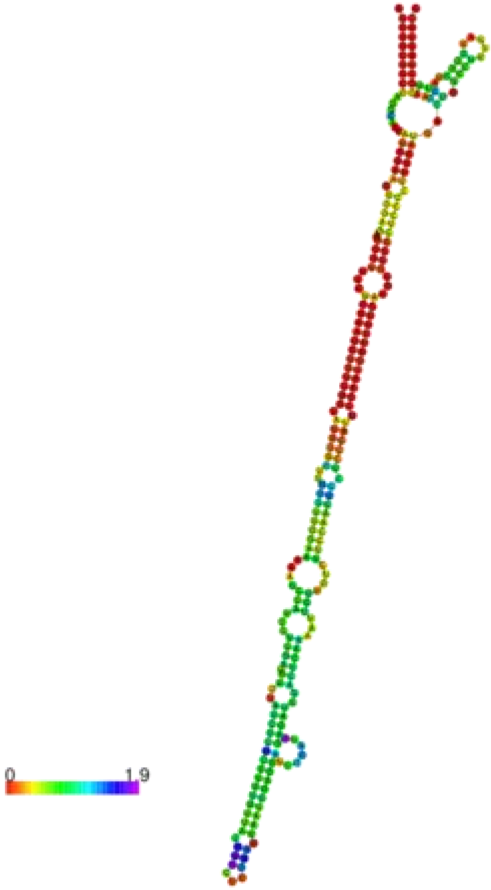

novel_mir221

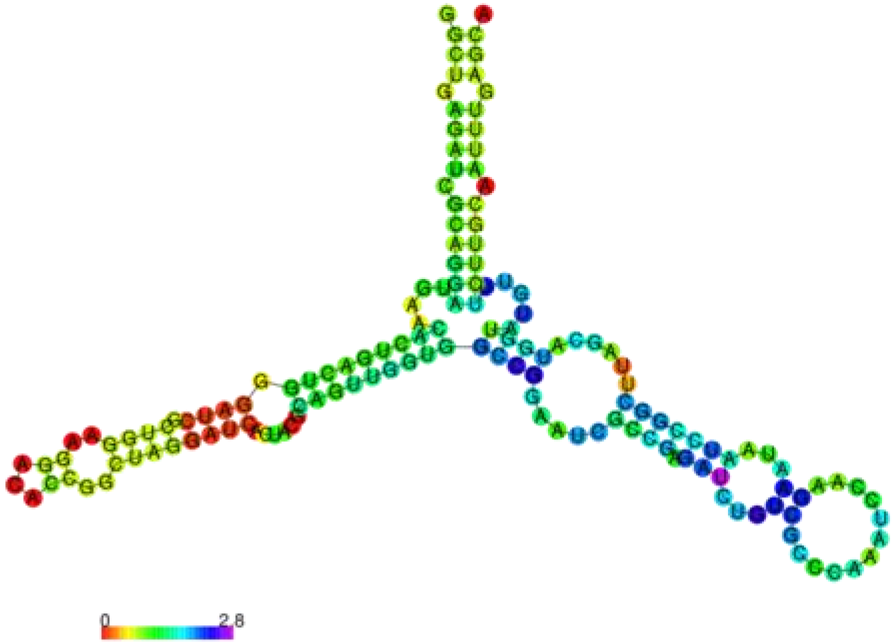

novel_mir222

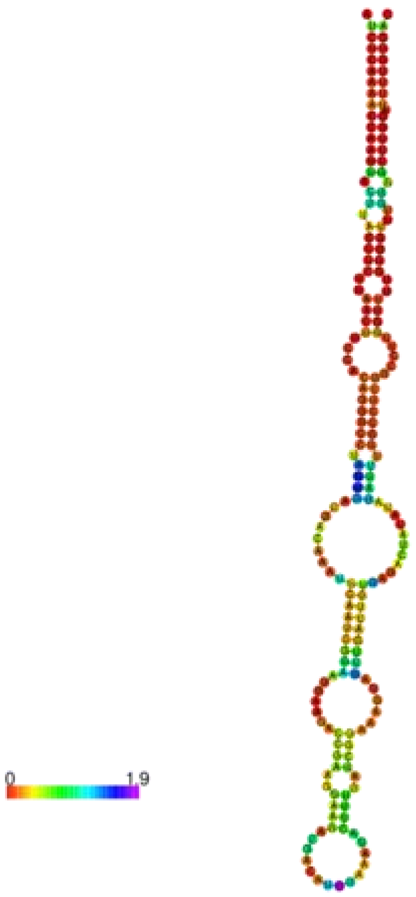

novel_mir224

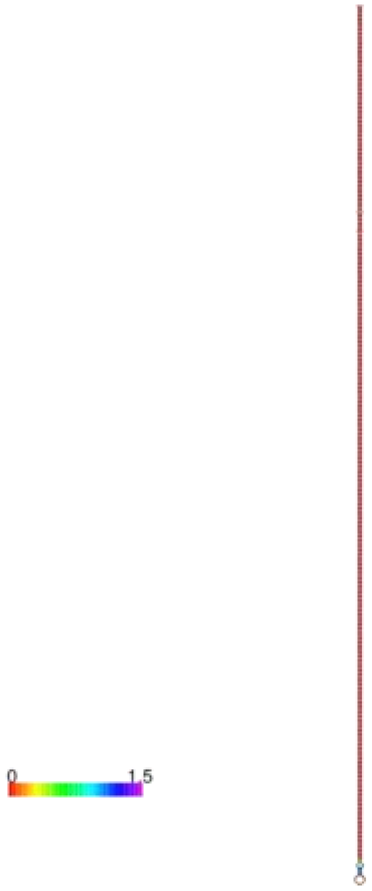

novel_mir23

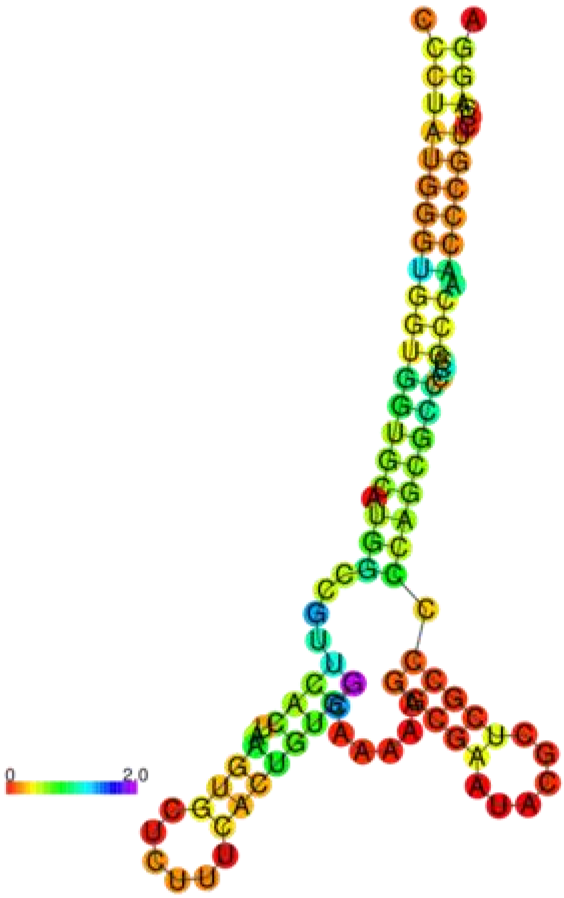

novel_mir231

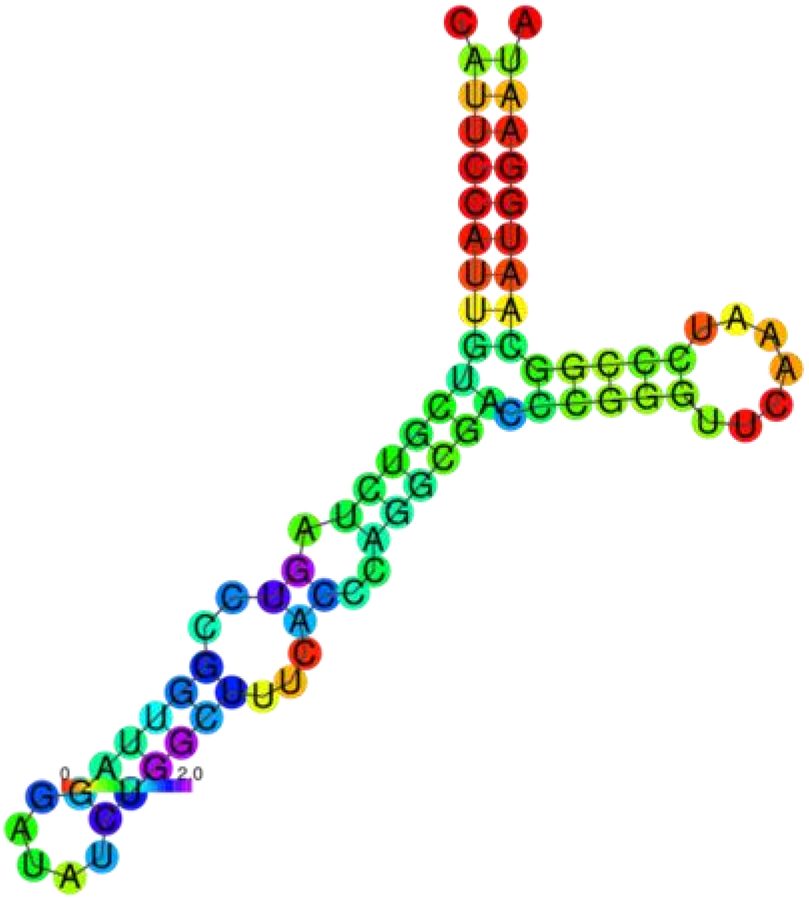

novel_mir234

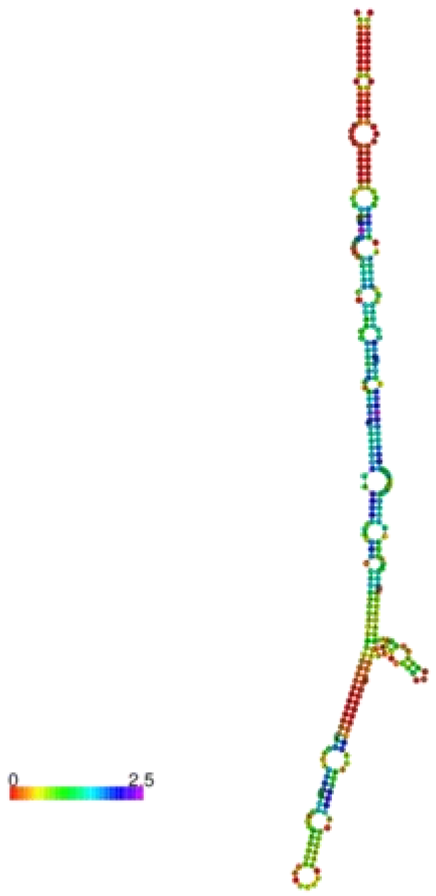

novel_mir236

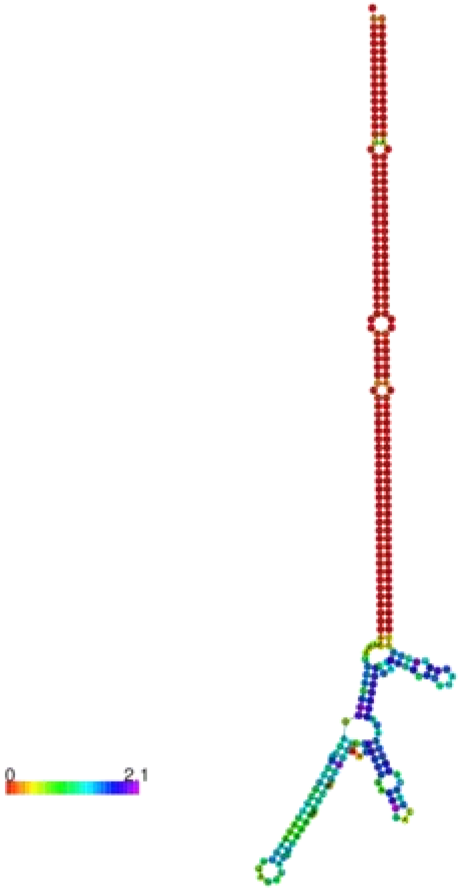

novel_mir238

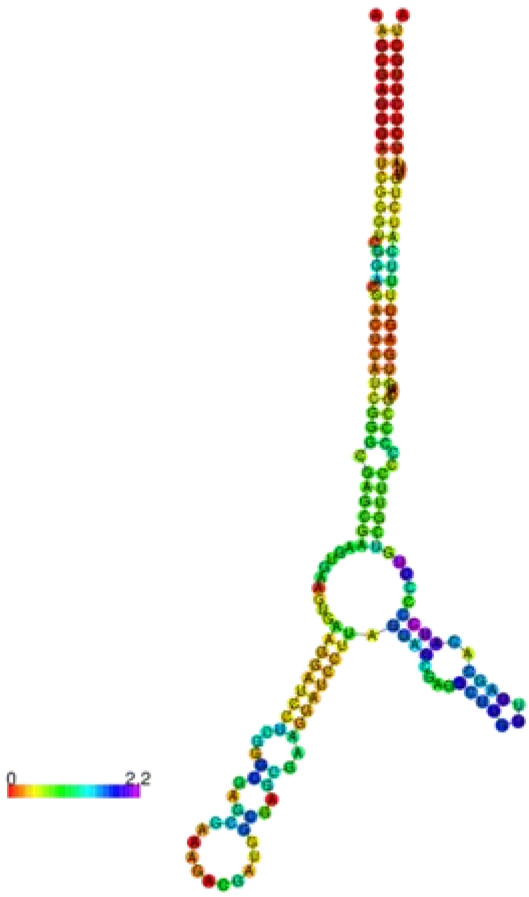

novel_mir239

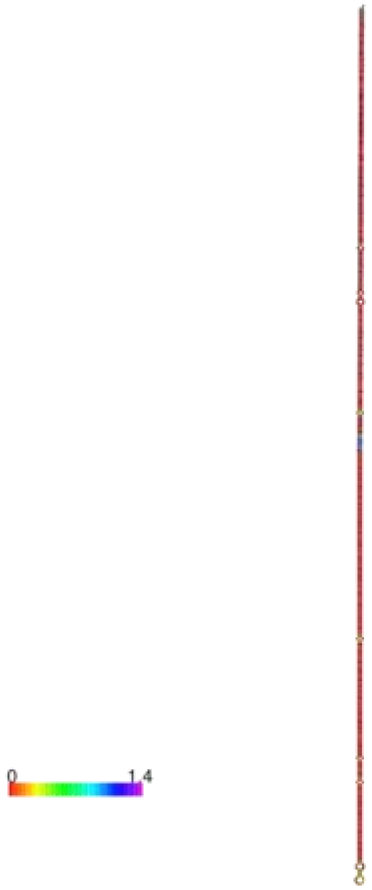

novel_mir242

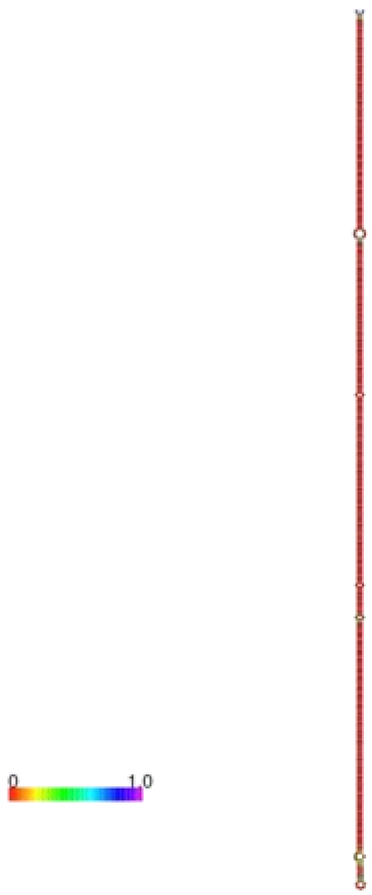

novel_mir3

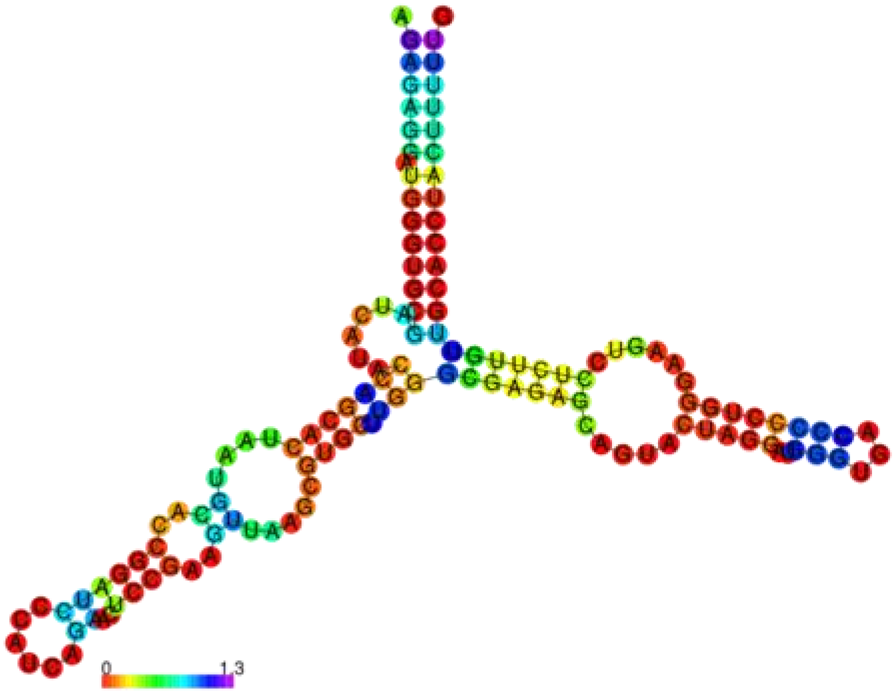

novel_mir31

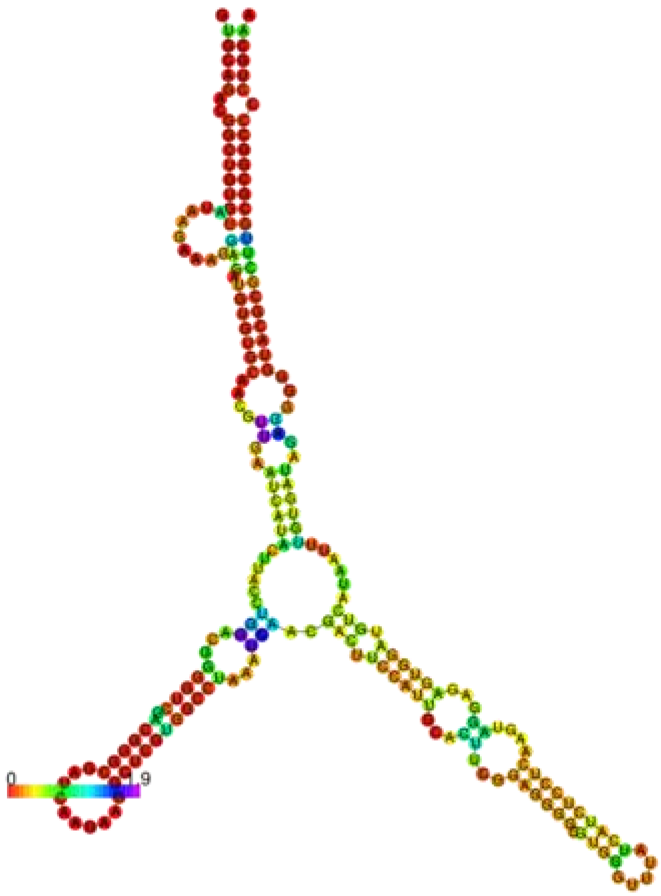

novel_mir32

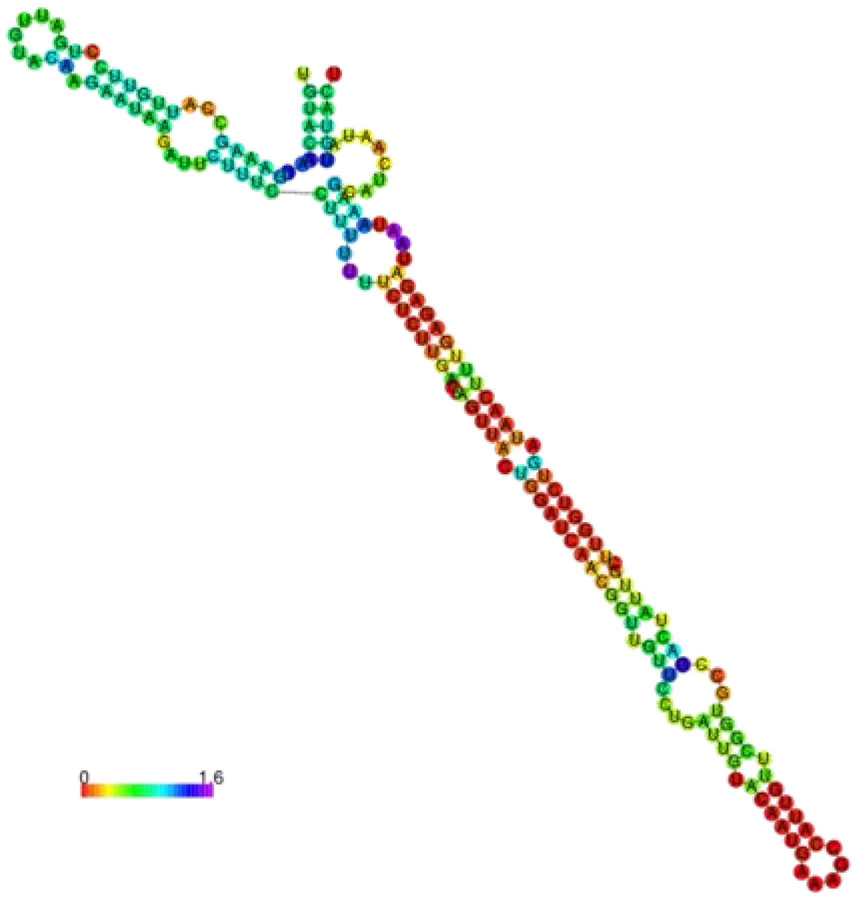

novel_mir35

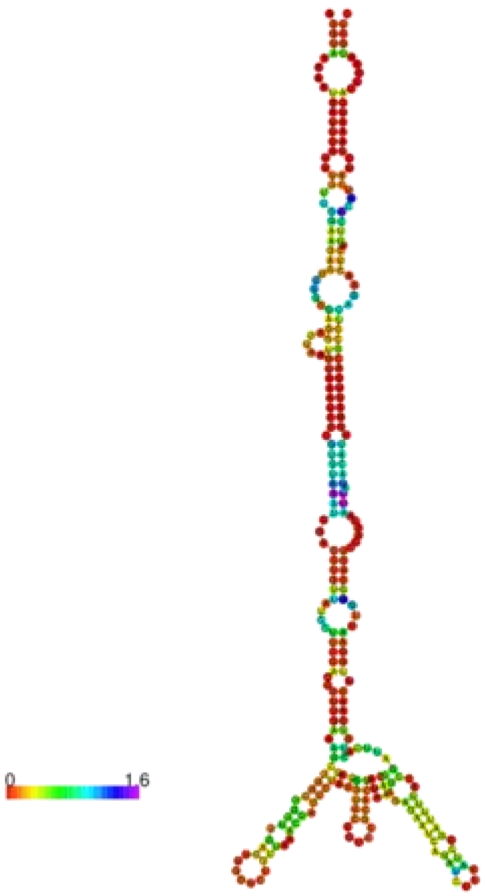

novel_mir38

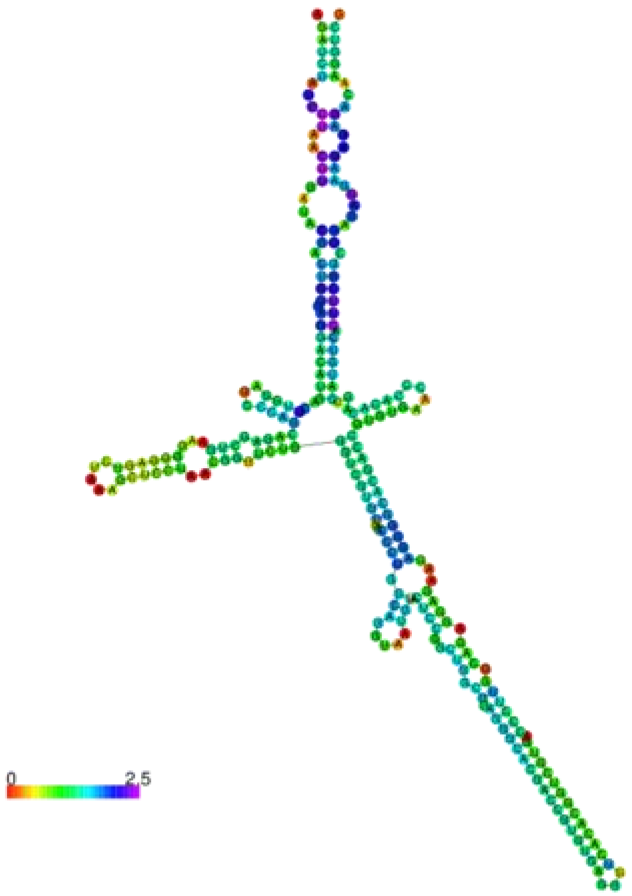

novel_mir4

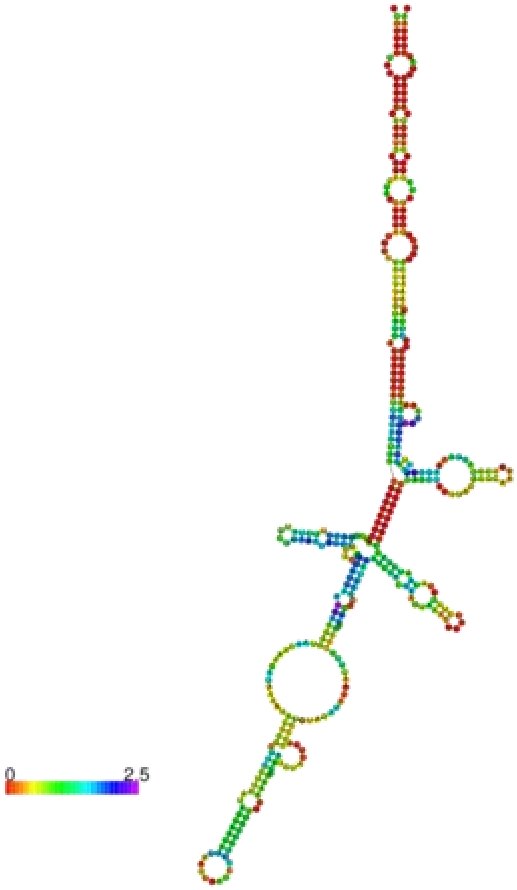

novel_mir42

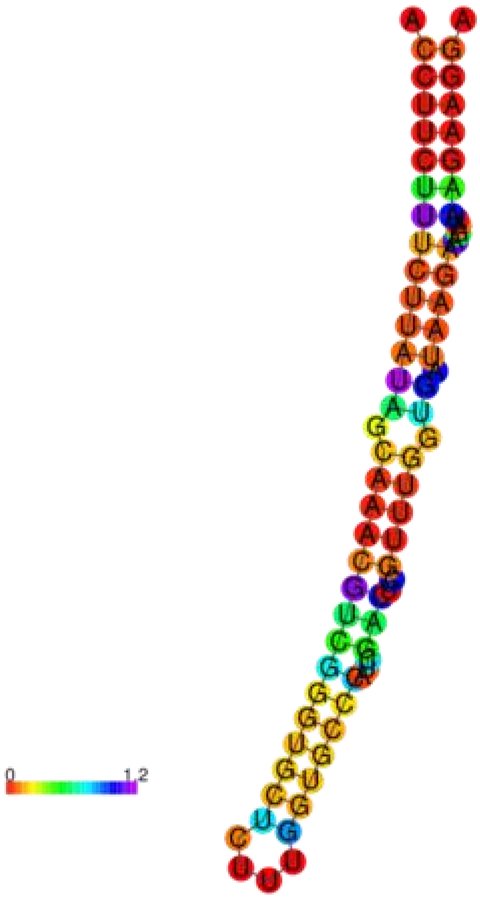

novel_mir47

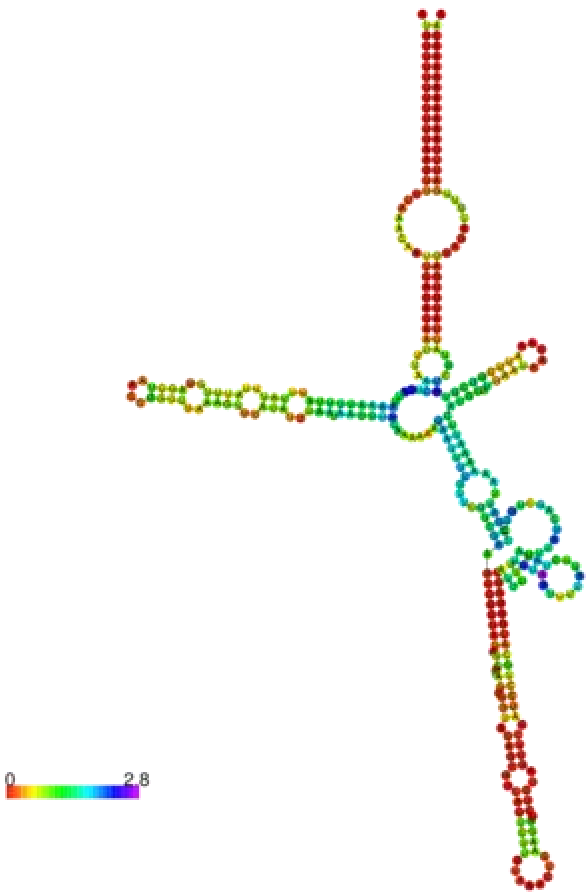

novel_mir5

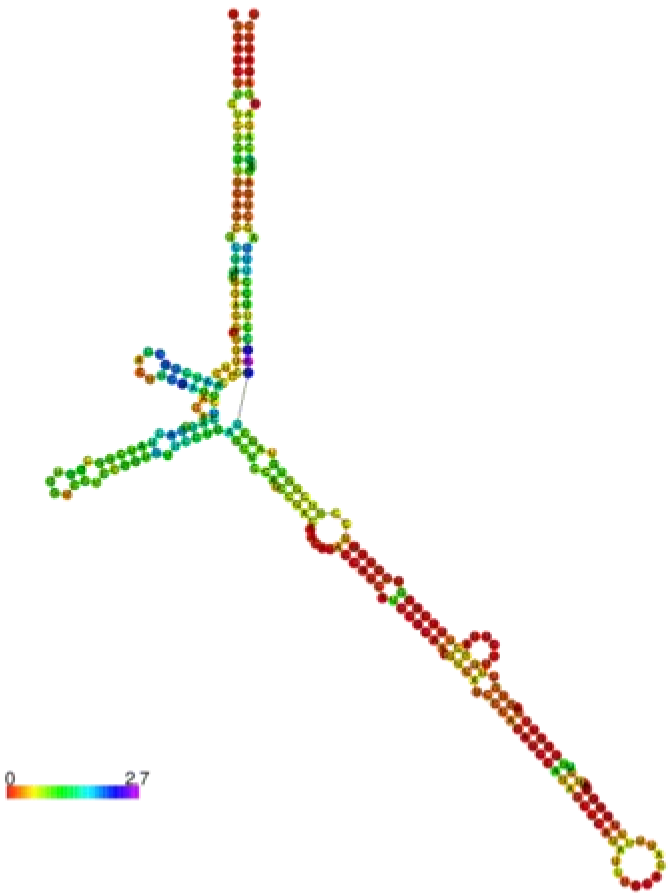

novel_mir51

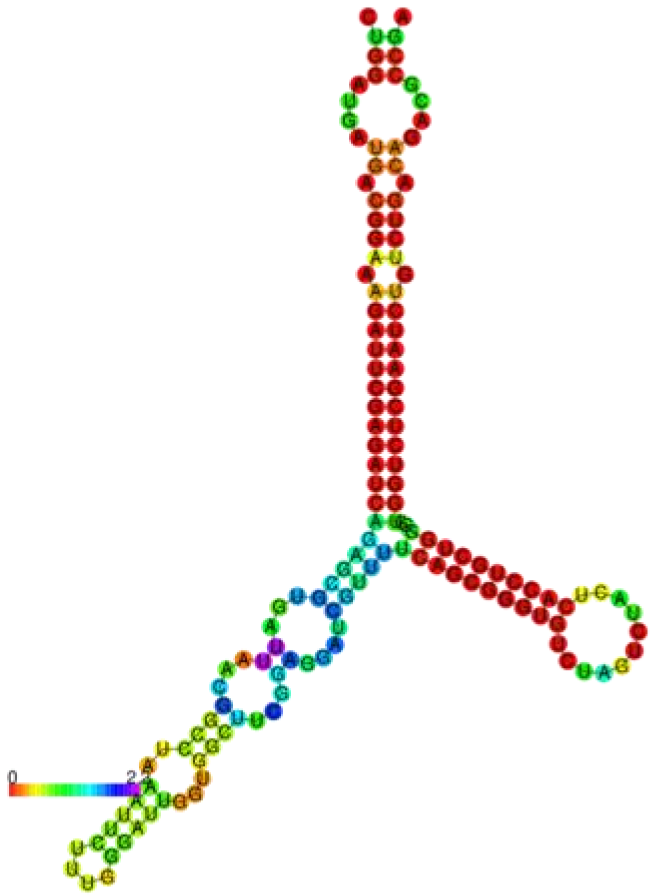

novel_mir52

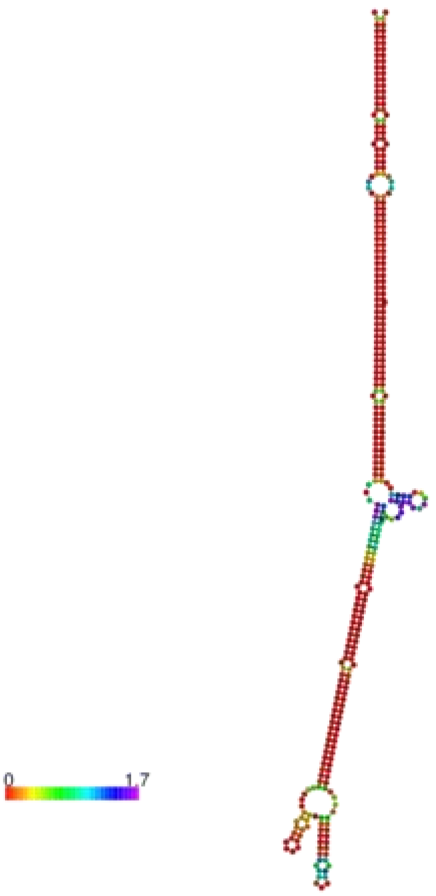

novel_mir53

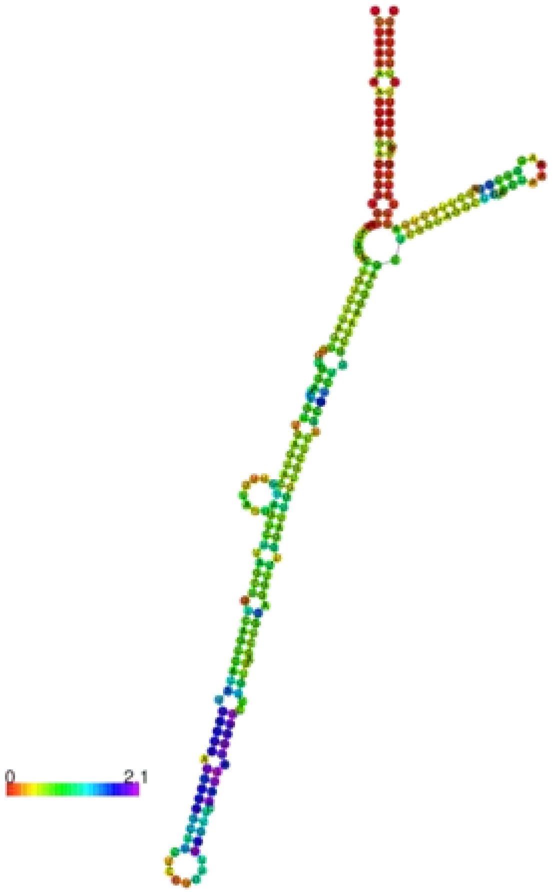

novel_mir54

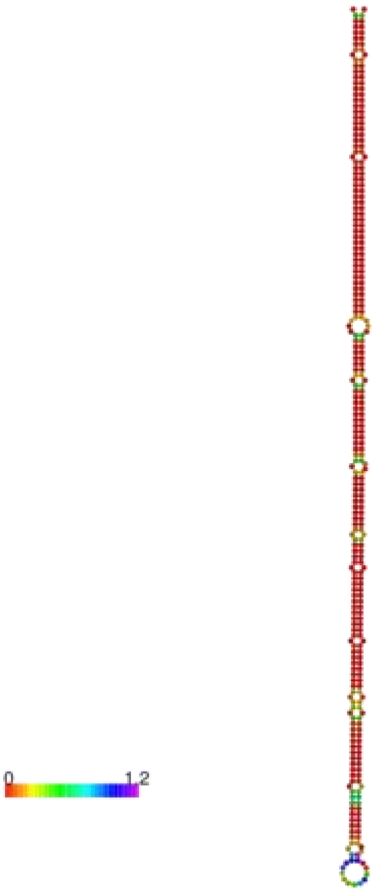

novel_mir58

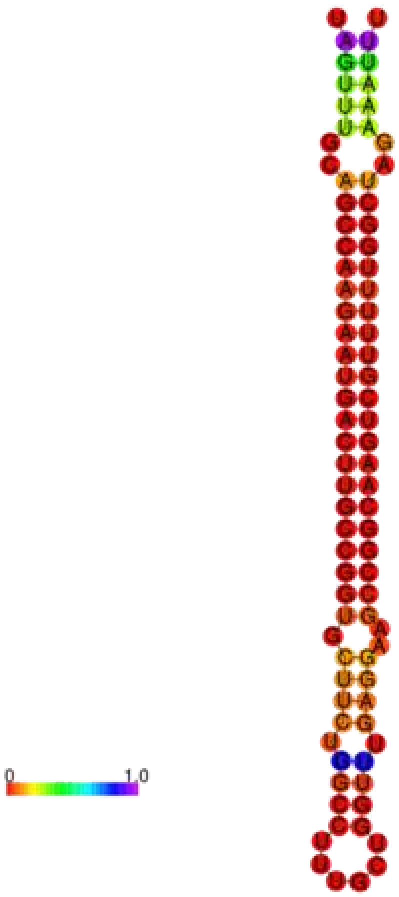

novel_mir59

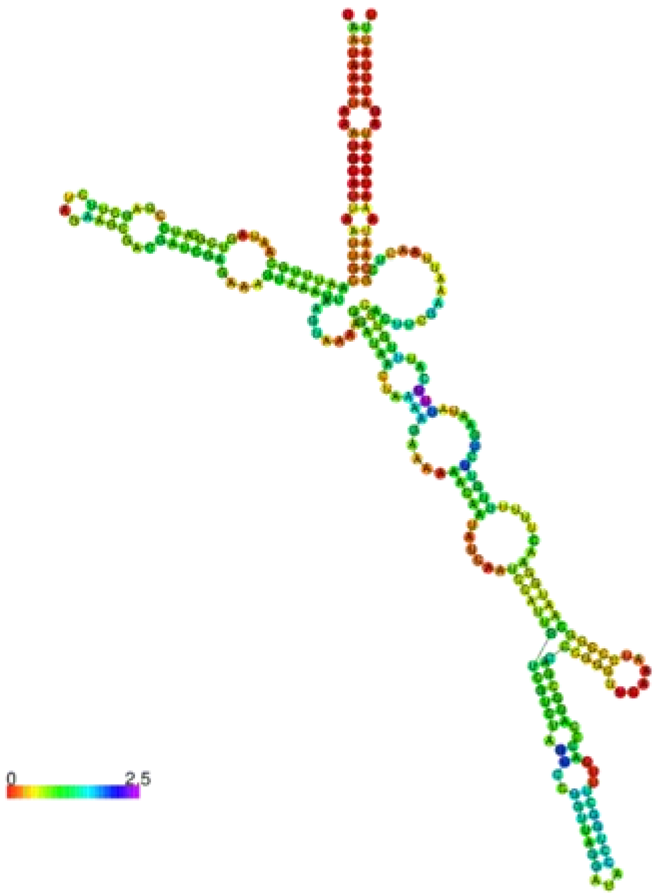

novel_mir60

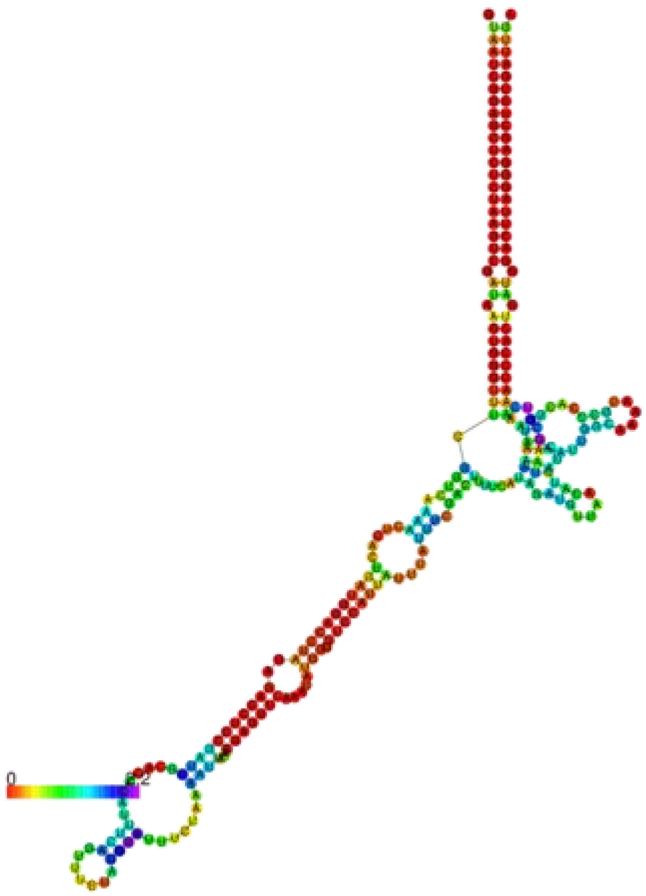

novel_mir62

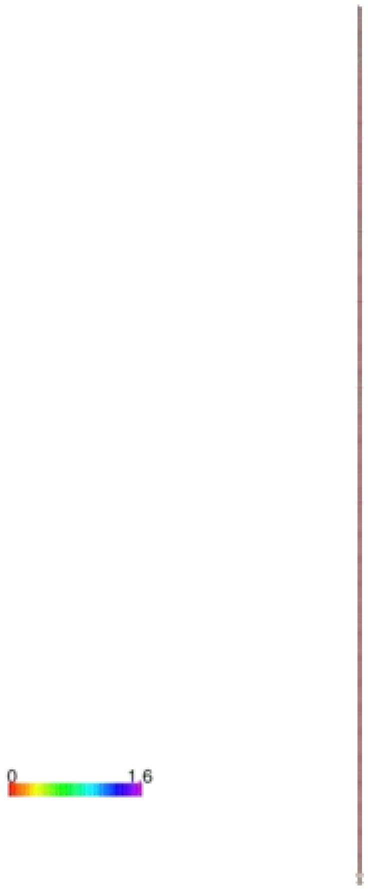

novel_mir64

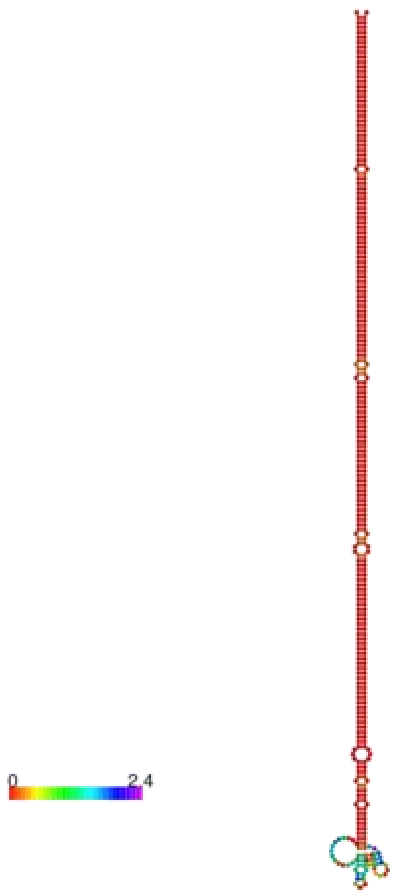

novel_mir67

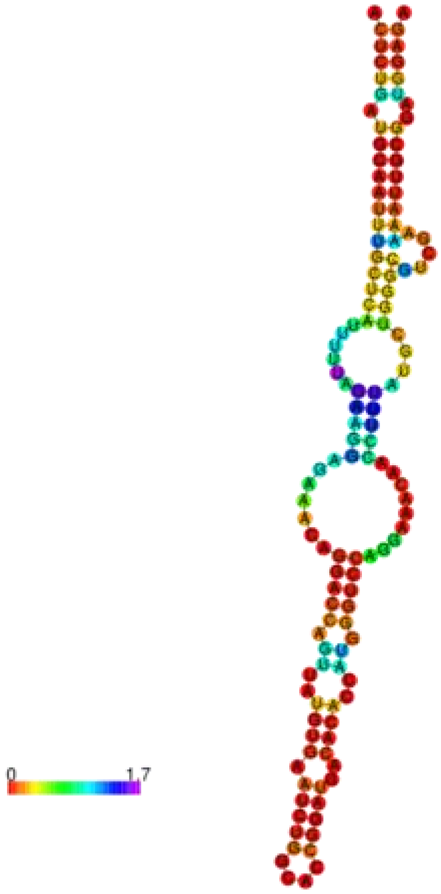

novel_mir7

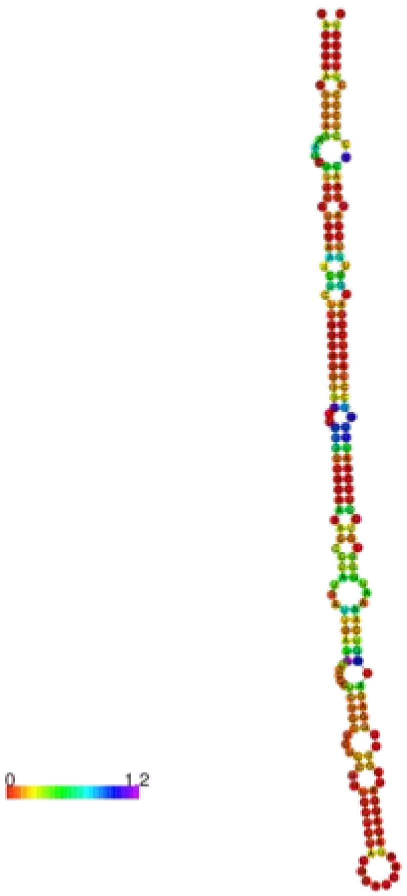

novel_mir71

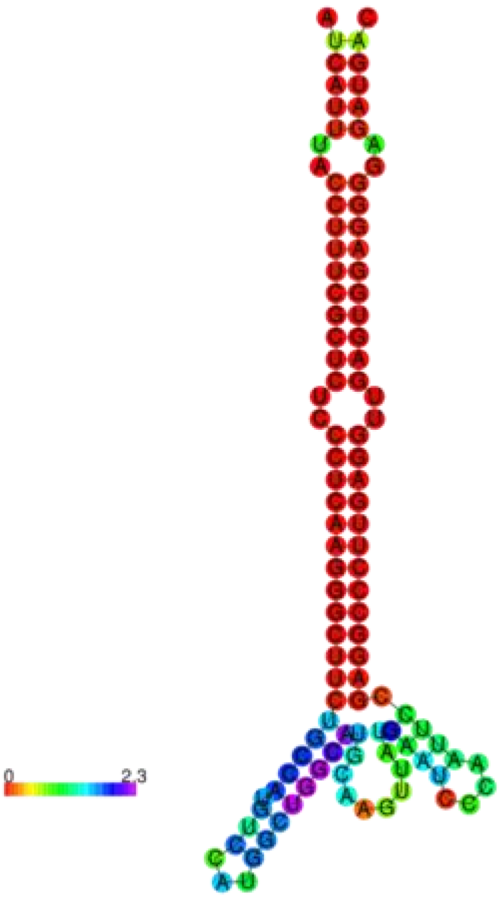

novel_mir78

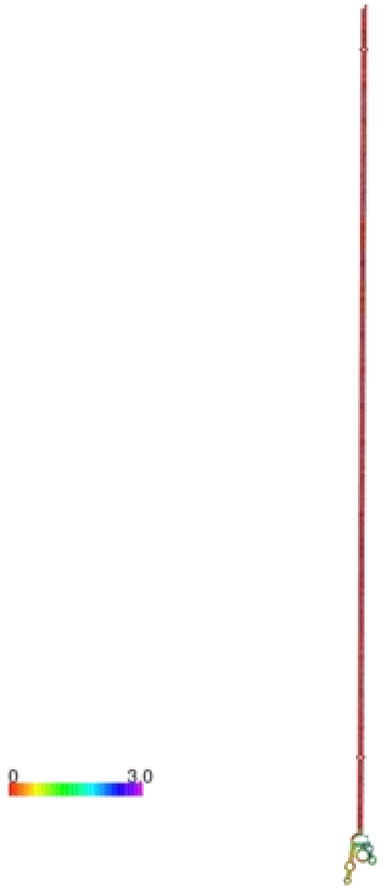

novel_mir84

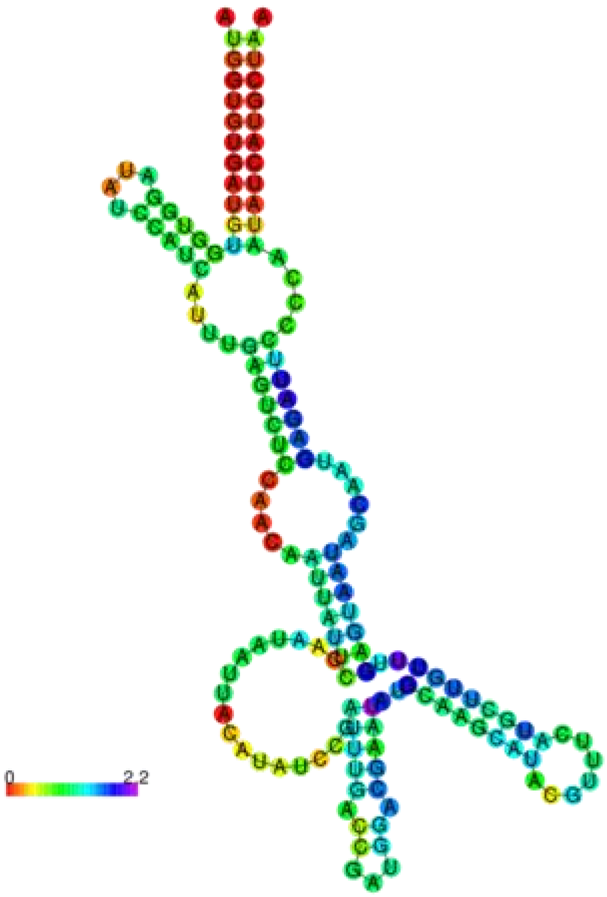

novel_mir9

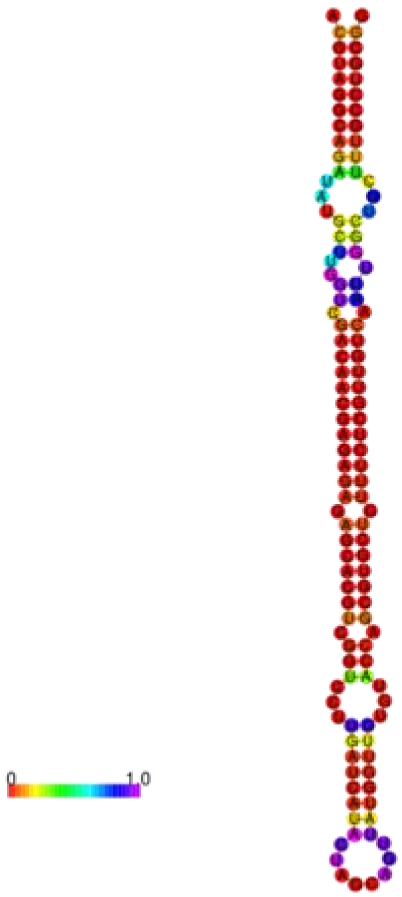

novel_mir90

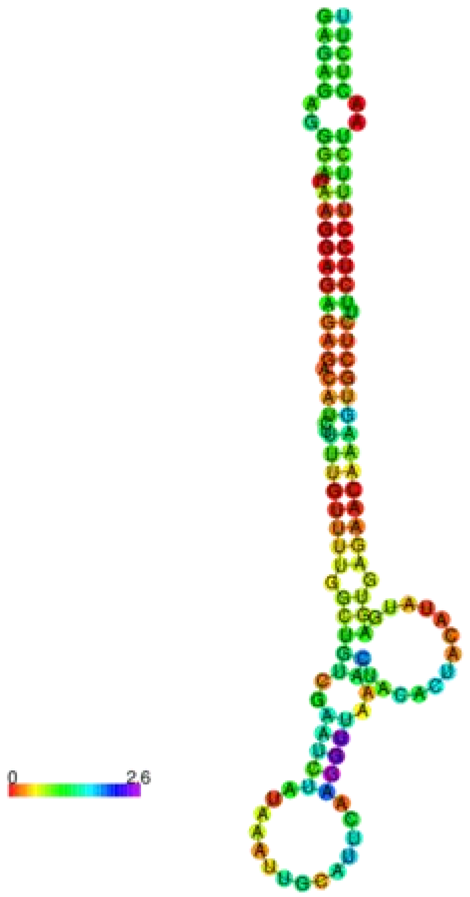

novel_mir92

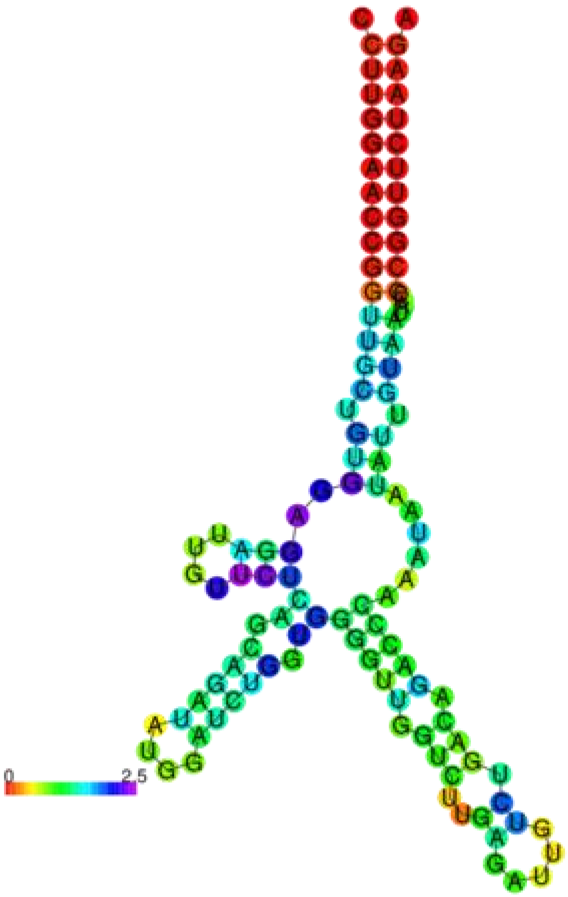

novel_mir96

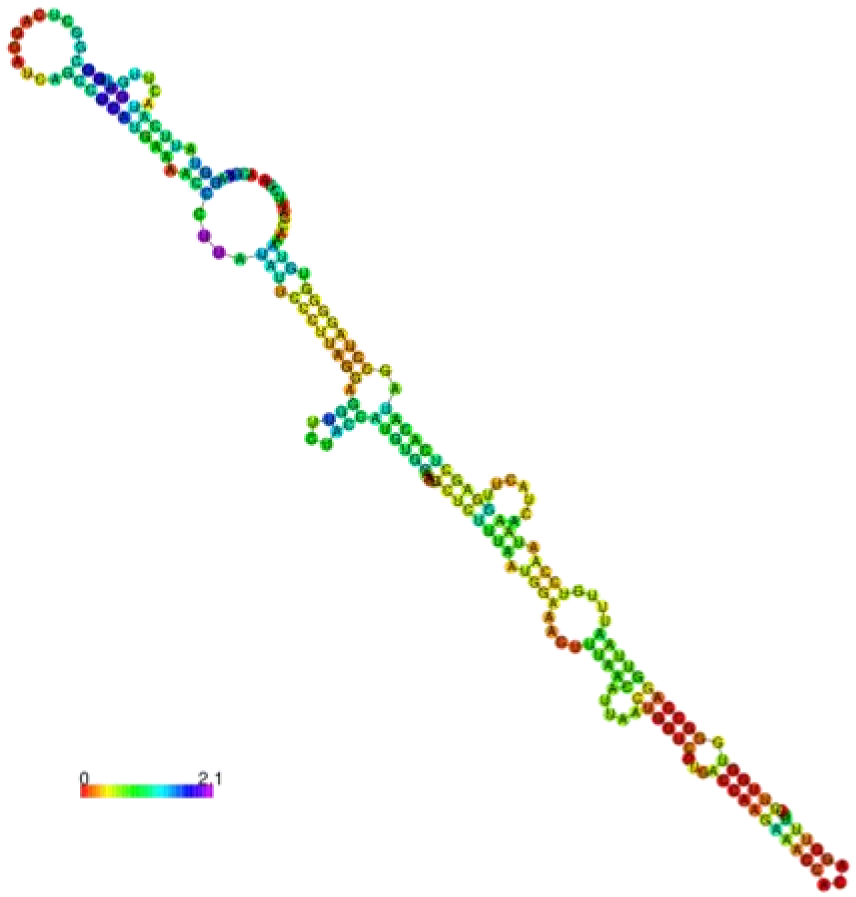

novel_mir97

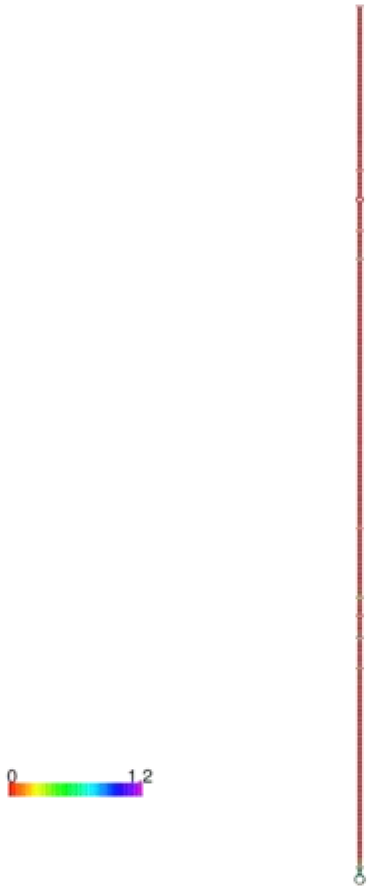

novel_mir99

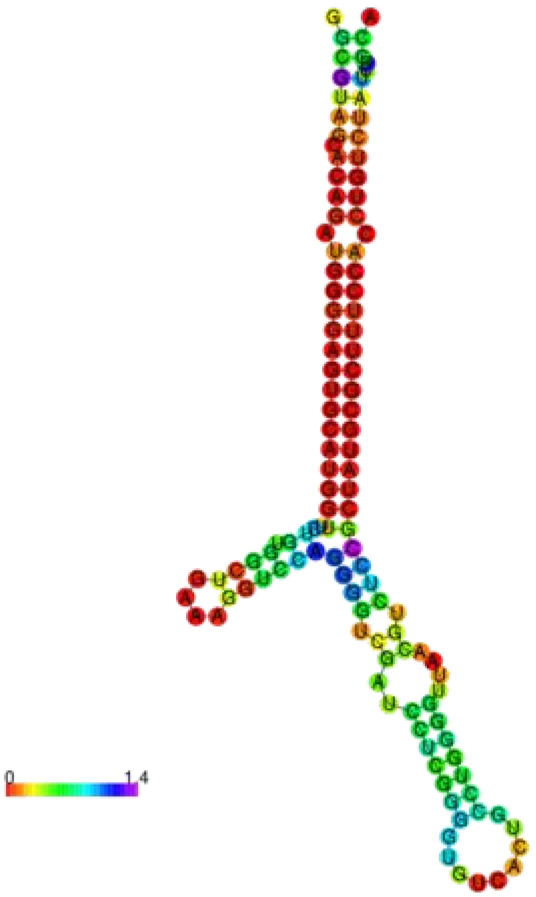
0
1.4
