# Supplementary material for: Genome-wide investigation of microRNAs and expression profiles during rhizome development in ginger (Zingiber officinale Roscoe)
Source: BMC Genomics. 2022 Jan 13;23:49. doi: 10.1186/s12864-021-08273-y (PMC8756691; doi:10.1186/s12864-021-08273-y)
Supplement: Supplementary file 5 — Additional file 5: Supplementary Figure S2. Expression profile of conserved miRNAs during rhizome development. Rz_1: Rhizome 1st I_d; Rz_2: Rhizome 2nd I_d; Rz_3: Rhizome 3rd I_d. [file 12864_2021_8273_MOESM5_ESM.pdf]

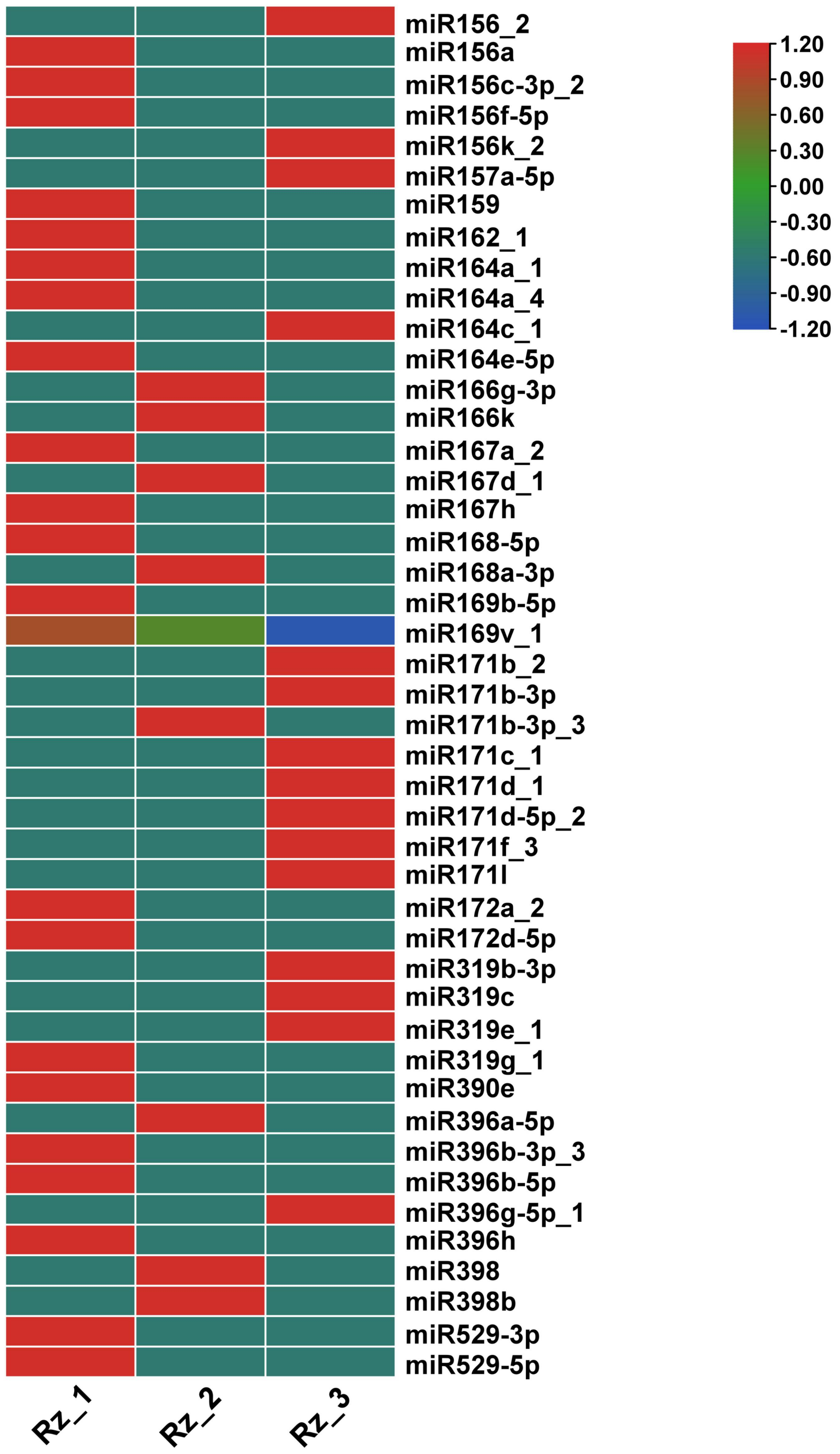

Supplementary Figure S2. Expression profile of conserved miRNAs during rhizome development. Rz_1: Rhizome 1st I_d; Rz_2: Rhizome 2nd I_d; Rz_3: Rhizome 3rd I_d.
